# Optimization and Kinetic Modeling of a Fed-Batch Fermentation for Mannosylerythritol Lipids (MEL) Production With *Moesziomyces aphidis*

**DOI:** 10.3389/fbioe.2022.913362

**Published:** 2022-05-17

**Authors:** Alexander Beck, Franziska Vogt, Lorena Hägele, Steffen Rupp, Susanne Zibek

**Affiliations:** ^1^ Institute of Interfacial Process Engineering and Plasma Technology IGVP, University of Stuttgart, Stuttgart, Germany; ^2^ Fraunhofer Institute for Interfacial Engineering and Biotechnology IGB, Stuttgart, Germany

**Keywords:** biosurfactants, ustilaginaceae, mannosylerythritol lipids, process engineering, bioreactor, kinetic modeling

## Abstract

Mannosylerythritol lipids are glycolipid biosurfactants with many interesting properties. Despite the general interest in those molecules and the need for a robust process, studies on their production in bioreactors are still scarce. In the current study, the fermentative production of MEL in a bioreactor with *Moesziomyces aphidis* was performed using a defined mineral salt medium. Several kinetic process parameters like substrate consumption rates and product formation rates were evaluated and subsequently enhanced by increasing the biomass concentration through an exponential fed-batch strategy. The fed-batch approaches resulted in two to three fold increased dry biomass concentrations of 10.9–15.5 g/L at the end of the growth phase, compared with 4.2 g/L in the batch process. Consequently, MEL formation rates were increased from 0.1 g/Lh up to around 0.4 g/Lh during the MEL production phase. Thus, a maximum concentration of up to 50.5 g/L MEL was obtained when oil was added in excess, but high concentrations of residual fatty acids were also present in the broth. By adjusting the oil feeding to biomass-specific hydrolysis and MEL production rates, a slightly lower MEL concentration of 34.3 g/L was obtained after 170 h, but at the same time a very pure crude lipid extract with more than 90% MEL and a much lower concentration of remaining fatty acids. With rapeseed oil as substrate, the ideal oil-to-biomass ratio for full substrate conversion was found to be around 10 g_oil_/g_biomass_. In addition, off-gas analysis and pH trends could be used to assess biomass growth and MEL production. Finally, kinetic models were developed and compared to the experimental data, allowing for a detailed prediction of the process behavior in future experiments.

## Introduction

The microbial glycolipids mannosylerythritol lipids (MEL) are biosurfactants produced by various fungi of the Ustilaginaceae family. Besides their surface activity and biodegradability, which make them suitable as fully bio-based surfactants for household and personal care applications, for example, MELs also possess some additional interesting properties. These are for example the ability to induce cell differentiation in mammalian cells (Isoda et al., 1997; Isoda and Nakahara, 1997; Wakamatsu et al., 2001), to interact with proteins or antibodies (Konishi et al., 2007; Fan et al., 2018), and to inhibit the growth of Gram-positive bacteria (Kitamoto et al., 1993; Liu et al., 2020; Shu et al., 2020). Moreover, they are reported to possess a moisturizing activity towards human skin (Morita et al., 2009b; Yamamoto et al., 2012) and hair (Morita et al., 2010a; Morita et al., 2010b). Their surface wetting ability could also make them suitable as agrochemicals (Fukuoka et al., 2015).

MEL has a hydrophilic core, 4-O-β-D-mannopyranosyl-D-erythritol, and several hydrophobic residues including two fatty acid chains at C2′ and C3′ and a different degree of acetylation at C4′ and C6’. They are traditionally classified according to their acetylation pattern into the four different congeners MEL-A, -B, -C and -D. MEL-A is the most hydrophobic congener, having two acetyl groups, while MEL-B and MEL-C are mono-acetylated at C6′ and C4’ respectively. MEL-D is not acetylated and is, therefore, the most hydrophilic variant. The chain length of the two fatty acid residues is highly species-specific and can range from a combination of C_10_-C_10_ to C_4_-C_16_. Some more unconventional and rare MEL variants, which can occur under specific process conditions or with genetically modified organisms, only have one fatty acid residue, so-called mono-acylated MELs (Fukuoka et al., 2007b; Saika et al., 2018; Saika et al., 2020), or an additional fatty acid, called tri-acylated MELs (Fukuoka et al., 2007a; Morita et al., 2008; Goossens et al., 2016; Beck et al., 2021).

The most efficient production processes for MEL use plant oils as a hydrophobic carbon source. Here, soybean, rapeseed and olive oil are among the most commonly employed substrates, but others have also been tested (see for example Beck et al. (2019b) or Morita et al. (2015) for a detailed overview). In theory, hydrocarbons like alkanes or alkenes are also possible substrates (Kitamoto et al., 2001), but they are non-renewable and thus rather for scientific purposes only. Hydrophilic carbon sources like sugars or glycerol can also be added to enhance biomass formation or MEL production, although MEL production solely from hydrophilic sources does not yield high MEL concentrations (Morita et al., 2009a; Faria et al., 2014).

The underlying metabolic pathway for MEL production has first been described in *Ustilago maydis* by Hewald et al. (2006). It comprises five essential steps that are catalyzed by respective enzymes. First, mannose and erythritol are linked by the erythritol-mannosyl-transferase Emt1. Secondly, two acylation reactions catalyzed by the acyltransferases Mac1 and Mac2 yield the basic molecule MEL-D. Selective acetylation by the acetyltransferase Mat one then generates the different congeners MEL-A, -B, -C and -D, before they are exported into the extracellular space by the transporter protein Mmf1 (Hewald et al., 2006). The necessary precursor molecules mannose and erythritol are generated from other sugars by glycolysis/gluconeogenesis, isomerization, and pentose phosphate pathway (Carly and Fickers, 2018; Masi et al., 2021). The two fatty acids are derived from the so-called chain-shortening pathway (Kitamoto et al., 1998), which is localized in cellular peroxisomes and produces fatty acids with the specific chain length (Freitag et al., 2014; Deinzer et al., 2019).

While many studies on MEL production in shake flasks, using different organisms and substrates, have been published over the last 3 decades, literature on dedicated process engineering in bioreactors is still scarce (see Beck et al. (2019b) for a detailed review). Only a few studies are dealing with MEL production in a bioreactor, and only some of them are presenting advances regarding process control and monitoring.

The first bioreactor process for MEL production was reported by Kim et al. (1999) using a 5-L glass fermenter. Process parameters for the batch cultivation with 100 g/L soybean oil were set at a temperature of 30°C, an aeration rate of one vvm (volume per volume per minute), and a stirring speed of 300 rpm. No parameter optimization was performed for this publication. Building on another study in which the process was optimized in shake flasks (Kim et al., 2002), Kim et al. (2006) later described a two-stage fed-batch fermentation using 30 g/L glucose and soybean oil (1:1 w/w) for growth and a subsequent feed of soybean oil (170 g/L in total) for MEL production. The fermentation in a 5-L bioreactor was conducted at a controlled dissolved oxygen level of 20% by varying stirrer speed (500–750 rpm) and aeration rate (0.two to two vvm) accordingly. With the optimized fed-batch approach, an increased MEL concentration of 95 g/L was obtained after 200 h, corresponding to a product yield coefficient Y_MEL/oil_ of 0.45 g/g and volumetric productivity of 0.48 g/Lh. In all of their publications, an isolate called *Candida* sp. *SY16* was used, which was identified as a *P. tsukubaensis* strain (Kim et al., 2006).

Adamczak and Bednarski (2000) highlighted the importance of proper aeration for the MEL production process in bioreactors. While sufficient oxygen supply was necessary for efficient biomass growth on the one hand, it could also lead to intensive foaming when biosurfactants were produced. Since the produced foam contained not only MEL but also cells and substrate lipids, foaming should be avoided in the MEL production process. The best MEL production of 46 g/L (after 144 h) with *M. antarcticus* was found to occur during batch cultivation with 80 g/L soybean oil at 30°C, a controlled oxygen level of 50% (100–500 rpm), and an aeration rate of one vvm. A two-stage process with glucose for growth and repeated soybean oil feeding was also investigated but led to decreased MEL concentrations of only 28 g/L (Adamczak and Bednarski, 2000).

Following up on these earlier publications, Rau et al. (2005a) realized the need for a more detailed investigation of bioreactor production to establish a more economic process. Based on a previous study of MEL production in shake flasks (Rau et al., 2005b), a fed-batch process in a 72-L bioreactor with *M. aphidis* was developed (Rau et al., 2005a). The idea was to use substrate feeding to further increase cell biomass. Stirring and aeration were manually adapted to minimize foaming. In their optimized fed-batch process 30 g/L glucose, 3 g/L sodium nitrate, and 20 ml/L soybean oil were used for batch growth, followed by feeding a concentrated growth solution (glucose, nitrate, and yeast extract). Further oil addition (∼126 g/L) was triggered by an anti-foam sensor every time foaming occurred. With the additional feeding of substrate, cell concentration was almost doubled. Overall, a MEL concentration of 165 g/L after 283 h was reported for the fed-batch process, which would correlate to a product yield Y_MEL/substrate_ of 0.92 g/g and overall volumetric productivity of 0.58 g/Lh (Rau et al., 2005a). The process of Rau et al. (2005a) as well as their culture medium is still one of the major references for MEL production processes and has been adapted by others, e.g. in Goossens et al. (2016).

A major drawback of all those published processes, in our opinion, is the fact that exclusively complex culture media with either peptone or yeast extract were employed. Besides being rather expensive, complex substrates such as yeast extract can generate problems during scale-up, such as lower batch-to-batch reproducibility, increased heat sensitivity during the sterilization process, and stronger foaming (Zhang and Greasham, 1999; Posch et al., 2012). Defined media, in turn, provide a better option for medium development and optimization, as all components and concentrations are known in detail. Therefore, we had established a novel defined mineral medium that was very effective for MEL production with several Ustilaginaceae species and especially *M. aphidis* in a previous work (Beck and Zibek, 2020a). Additionally, a positive correlation between biomass concentration at the end of the growth phase and subsequent oil conversion into MEL was observed in that publication, indicating that higher MEL concentrations and higher MEL production rates can be obtained when the active biomass concentration is increased during the initial cell growth phase.

With the current work, we further followed this path and successfully established a stable fermentation process with high controllability in an aerated stirred-tank bioreactor for the growth of *M. aphidis* and subsequent MEL production. The fermentation medium was based on our novel defined mineral medium (Beck and Zibek, 2020a), with glucose and rapeseed oil as carbon substrates for growth and production, respectively. The composition of the mineral medium was first characterized in terms of C/N, C/P and C/S ratios to determine the ideal ratios for efficient biomass growth and MEL production. Alternative sugar substrates for growth were also investigated. In the stirred-tank bioreactor, several process variations with either batch or fed-batch growth phase as well as different amounts of oil feeding were then examined. One aim was to demonstrate that a fed-batch growth phase led to higher biomass concentrations and subsequently faster MEL production with higher concentrations compared to the batch process. Secondly, the amount of plant oil feeding during the production phase was adapted to the biomass concentration at the end of the growth phase to enable complete conversion of the plant oil and fatty acids into MEL. This was done to use the oil substrate as efficiently as possible and to achieve a high purity of the crude lipid extract at the end of the fermentation process. This in turn facilitates subsequent downstream processing, where MEL has to be separated from excess lipids. During all these experiments in the bioreactor, key parameters like formation rates and yields for biomass and product, as well as specific oxygen requirements were systematically determined for the first time. These process values were ultimately used to create a kinetic model that adequately described the time course of the fermentations and can be exploited for further scale-up studies.

## Materials and Methods

### Microorganism

*Moesziomyces aphidis* DSM 70725, obtained from the German Collection of Microorganisms and Cell Cultures (DSMZ; Braunschweig, Germany), was used for MEL production in all experiments.

### Chemicals, Culture Media and Substrates

All chemicals were obtained from either Th. Geyer (Renningen, Germany), Merck (previously Sigma-Aldrich, Darmstadt, Germany), or Carl Roth (Karlsruhe, Germany) unless specified otherwise.

For maintenance of the microorganism, potato dextrose (PD) agar slants were used. They were prepared with 24 g/L potato dextrose broth (Bacto TM, Becton, United States) and 20 g/L agar.

Liquid culture medium for seed-culture and stirred-tank bioreactor fermentations consisted of 30 g/L glucose, 3 g/L NaNO_3_, 1 g/L KH_2_PO_4_, 1 g/L MgSO_4_*7H_2_O, 1.1 g/L KCl, 0.15 g/L CaCl*2H_2_O as well as a vitamin and trace element solution, with an initial pH 5.5 (not adjusted) (Beck and Zibek, 2020a). Vitamin and trace element solutions were prepared as described in this previous publication, filter-sterilized, and supplemented to the bioreactor. For the fed-batch processes in the bioreactor, a concentrated feeding solution with 300 g/L glucose and 30 g/L NaNO_3_ was used. Other salts, trace elements, and vitamins were the same concentration as in the batch medium.

Commercial food-grade rapeseed oil from a local supermarket (Kaufland, Germany) was used as the inductor and main carbon substrate for the MEL production phase. The oil was autoclaved separately and fed to the fermenter using a feeding flask and pump.

For the investigation of alternative sugar sources for growth, crystalline sugars like glucose, sucrose, fructose, xylose, arabinose, and cellobiose were bought from Carl Roth (Karlsruhe, Germany). Moreover, intermediate or side-streams from a sugar refining plant were kindly provided by Pfeifer & Langen Industrie-und Handels-KG (Köln, Germany). These were syrup, sugar beet molasses, sugar cane molasses, as well as two different process waters A and B. Except for process water B, which had a total sugar content of only 44 g/kg and contained mostly fructose and glucose, all other fractions had a sugar content of 62–74 g/kg and contained predominantly sucrose.

### Seed Cultivation for Inoculum Preparation

Cryo-cultures of *M. aphidis* were thawed, plated onto PD agar plates and incubated at 30°C for 72 h. The inoculum for bioreactor fermentations was prepared in a two-step procedure, first in 100-ml and then in 1-L baffled shaking flasks. In order to ensure sufficient oxygen transfer for fast cell growth, the working volume was set to 20% v/v, i.e. 20 or 200 ml liquid culture medium respectively. The first seed culture was inoculated with a single loop from the agar plate and incubated for 72 h at 30°C and 110 rpm to disperse and grow the cells and to achieve a homogenous suspension. The second seed culture was then inoculated with 10–20 ml of the first seed culture to a defined initial OD_625_ of 0.7 and incubated at 30°C and 110 rpm for around 24 h, until an OD_625_ > 6 was reached so that sufficient inoculum was available for fermentation.

### Microreactor Cultivations

Screening experiments for medium composition and alternative carbon substrates were done with the BioLector I (m2p Labs GmbH, Baesweiler, Germany) microcultivation system. Cultivations were performed in flower-shaped 48-well microtiter plates (m2p Labs GmbH, Germany) sealed with adhesive gas-permeable and evaporation-reduced membranes (m2p Labs GmbH, Germany). Non-invasive online measurements of scattered light, pH and dissolved oxygen (DO) levels were recorded to assess the growth behavior as previously reported (Beck and Zibek, 2020a). The wells were filled with 1,000 µL culture medium and inoculated to an initial OD_625_ of 0.6, with the same seed cultivation procedure as for the bioreactor. Temperature and humidity in the system were controlled at 30°C and 85% relative humidity and the plates were shaken at 1,100 rpm with a diameter of 3.0 mm. Cycle time was 10 min, allowing for sequential measurement of backscatter (gain 5 and 20), pH and DO. Like in the bioreactor, MEL production was initiated after the cease of growth at around 48 h using 8% v/v of rapeseed oil (80 µL) in order to investigate the influence of growth phase on MEL production. Total process duration was 210 h.

The experimental design for the evaluation of medium composition - with regard to C/N, C/P, and C/S ratios - was based on a randomized Box-Behnken design with three factors and was created with DesignExpert 13 software (StatEase, Minneapolis, United States). The center point was performed as triplicate. This resulted in a set of 15 experiments. Two additional runs at the corners of the design space were conducted for model verification. The input variables were concentrations of NaNO_3_ (3–6 g/L), KH_2_PO_4_ (1–2 g/L) and MgSO_4_*7H_2_O (1-2 g/L) at a fixed glucose concentration of 30 g/L, leading to molar ratios of the respective elements (mol/mol) of 28.4–14.2 (C/N), 136-68 (C/P) and 244-122 (C/S). Output variables were biomass concentration at the end of growth (measured online as backscatter) and MEL concentration, which was quantified after the proceeding production phase on rapeseed oil (210 h process time). The 15 + 2 experiments were conducted simultaneously in a randomized order in the microcultivation system. Evaluation of the results by ANOVA was done with DesignExpert 13.

For the screening of alternative sugar sources, the experiments were performed as biological triplicates in the microcultivation system. The different carbon substrates were employed at the same total sugar concentration of 30 g/L. After the growth phase was terminated, one well of each sugar triplicate was harvested for offline analysis of sugar content, and the remaining two wells were fed with rapeseed oil to investigate a possible influence of the different sugars on subsequent MEL production.

### Stirred-Tank Bioreactor (STBR) Fermentation

Fermentations in the aerated stirred-tank bioreactor (STBR) were performed in a 7-L Labfors bioreactor (Infors HT, Bottmingen, Switzerland) equipped with two rushton turbines (d = 54 mm), four baffles and probes for online measurement and control of temperature, pH and DO. The bioreactor was filled with either 4 L (batch growth) or 3 L (fed-batch growth) culture medium and inoculated with 200–400 ml of the second seed culture to an initial OD_625_ of 0.6. Temperature was controlled at 30°C. The pH was maintained at pH 6 throughout the fermentation using H_2_SO_4_ (1 M) and NaOH (4 M). Aeration was set at 0.7 vvm and the dissolved oxygen concentration was controlled by dynamic stirrer speed adjustment between 400–1,200 rpm to maintain a DO of 10%. Foaming was controlled by mounting mechanical foam breakers onto the stirrer shaft in the headspace of the reactor. Off-gas concentrations of O_2_ and CO_2_ were measured using BlueSens BCP off-gas sensors (BlueSens GmbH, Herten, Germany).

The process consists of two separate phases: a biomass growth phase with glucose as carbon source and other nutrients, as well as a subsequent MEL production phase where rapeseed oil was added after glucose depletion. The growth phase could further be divided into an initial batch growth phase and a subsequent fed-batch phase where a concentrated solution of carbon source, nitrogen source and nutrients was fed continuously. This feeding was done using the built-in programmable feeding pump of the Labfors bioreactor. The pump was calibrated for different volumetric flow rates. During fed-batch phase, an exponentially increasing flow rate was used, which was calculated from the desired specific growth rate (µ_set_) according to the following equation (Chmiel et al., 2018):

$$F_{in}\left(t\right)={\left(V_{L}\;c_{x}\right)}_{0}\frac{{µ}_{set}}{Y_{X/gluc}\;\left(c_{gluc,Feed}-c_{gluc}\right)}{exp}^{\left({µ}_{set}\;t\right)}$$ (1)

Specific growth rates µ_set_ were set at either 0.08 or 0.09 h^−1^. Other parameters like biomass concentration at the end of the batch growth and biomass yield coefficient were derived from previous experiments (Beck and Zibek, 2020a) and set at c_x,0_ = 7 g/L and Y_X/gluc_ = 0.25 g/g initially.

Finally, the MEL production phase was initiated at the end of the growth phase by adding a defined amount of rapeseed oil to the fermenter. Within the production phase, individual process times and oil feeding strategies were studied to investigate the best feeding conditions (see results section).

### Sampling and Analytics

For off-line analysis of optical density, dry biomass, glucose and nitrate concentrations in the bioreactor, samples of 7 ml were drawn at regular intervals and treated as follows:

OD_625_ was measured in duplicates in a photometer (Gilson Inc., Middleton, United States) after appropriate dilution. For the quantification of dry biomass, samples of 1–5 ml, depending on the expected cell mass, were pipetted onto pre-dried and weighed filters and washed multiple times with water and ethanol to remove salts and residual substrates. After drying at 110°C for 24 h, the filters were weighed again and dry biomass was calculated from the weight difference and volume. The identical method was used to prepare samples for elemental analysis of the biomass. A correlation of dry biomass with OD_625_ values showed a linear trend during growth phase, where dry biomass [g/L] = 0.35 * OD_625_ [-]. Moreover, backscatter values obtained in the micro-cultivation system were correlated as well, showing a linear trend of backscatter gain 5 [-] = 0.14 * OD_625_ [-].

For determination of carbohydrates and sodium nitrate concentration, 1 ml of the sample was centrifuged for 10 min at 16,000 g and the supernatant was collected. An aliquot of the supernatant was diluted with 5 mM H_2_SO_4_, filtered with 3 kDa polyethersulfone (PES) centrifugal filters and measured by HPLC (Bischoff GmbH, Germany). Separation of the analytes was performed on a Phenomenex Rezex ROA-Organic Acid H+ (8%) column (30 cm × 7.8 mm, Phenomenex, Aschaffenburg, Germany) with 5 mM H_2_SO_4_ as mobile phase at a flow rate of 0.6 ml/min and 30°C column temperature. Detection of the sugars was done with a refractive index (RI) detector (Bischoff GmbH, Germany). External standards of D-glucose, D-mannose, D-mannitol, erythritol and glycerol (0.125–10 g/L each) were used for calibration. For NaNO_3_ quantification, the supernatant was diluted with de-ionized water and analyzed with an enzymatic nitrate reductase test (R-Biopharm, Darmstadt, Germany) in a miniaturized 96-well-plate format. External NaNO_3_ standards between 0.02–0.3 g/L were used on each plate for calibration.

For analysis of hydrophobic substances like triglycerides, fatty acids and MEL, 800 µL of culture broth was extracted with 800 µL of ethyl acetate by shaking at 1,400 rpm for 15 min, followed by centrifugation at 16,000 g and room temperature for 5 min to separate the two phases. Of the organic supernatant, 500 µL were collected and the solvent evaporated until constant weight. After weighing the crude lipid extract, it was re-suspended with pure ethanol to a total concentration of 20 g/L crude extract and analyzed by high-performance thin-layer chromatography (HPTLC). HPTLC was performed on HPTLC silica 60 plates (20 × 10 cm, Merck, Germany) using a solvent system consisting of chloroform-methanol (20:3 v/v). A sample volume of 2 µL was spotted with an ATS4 automatic TLC sampler (CAMAG, Muttenz, Switzerland). After development, the HPTLC plates were stained by dipping for one second into acetic acid/p-anisaldehyde/sulphuric acid (97:1:2 v/v/v) reagent solution, heated to 110°C and quantified densitometrically with the gel analyzer function of ImageJ. Calibration of MEL concentration between 2 and 20 g/L was done with a representative purified MEL standard from a previous *M. aphidis* cultivation, which was purified as published previously (Beck et al., 2019a). Fatty acid and residual oil concentrations were calibrated using oleic acid and commercial rapeseed oil as external standards in the range from 1–10 g/L.

### Data Analysis and Calculations

Online parameters like pH, temperature T, dissolved oxygen concentration DO, stirrer speed n and the respective volumes of pH-correcting agents and feeding solution were logged by Iris fermentation software (Infors HT, Bottmingen, Switzerland). Off-gas concentrations for oxygen O_2_ and carbon dioxide CO_2_ were logged with FermVis software (BlueSens GmbH, Herten, Germany).

In addition to the parameters that were directly measured online or offline, other key parameters for fermentation evaluation and modeling like volumetric and biomass-specific rates as well as yields were analyzed. All equations are derived from generally accepted growth kinetics (Chmiel et al., 2018).

The specific growth rate µ was calculated from the difference in dry biomass concentration c_x_:

$$µ=\frac{1}{c_{x}}\frac{\text{Δ}c_{x}}{\text{Δ}t}$$ (2)

Volumetric substrate consumption rates r_S_, yield coefficients Y_X/S_ and biomass-specific substrate consumption rates q_S_ were determined for glucose and nitrate respectively:

$$r_{S}=\frac{\text{Δ}c_{S}}{\text{Δ}t};Y_{X/S}=\frac{\text{Δ}c_{x}}{\text{Δ}c_{S}};q_{S}=\frac{r_{s}}{c_{x}}=\frac{1}{c_{x}}\frac{\text{Δ}c_{S}}{\text{Δ}t}=\frac{1}{Y_{X/S}}\;µ$$ (3)

Oxygen uptake rates (OUR), carbon dioxide emission rates (CER) and respiratory coefficients (RQ) were calculated from in- and off-gas concentrations y_O2_ and y_CO2_ (*α* = gas inlet, ω = exhaust) based on the following formula:

$$OUR=r_{O2}=\frac{Q_{air}p_{air}}{V_{L}\;R\;T}*\left(y_{O2}^{\propto }-\frac{1-y_{O2}^{\propto }-y_{CO2}^{\propto }}{1-y_{O2}^{\omega }-y_{CO2}^{\omega }}y_{O2}^{\omega }\right)$$ (4)

$$CER=r_{CO2}=\frac{Q_{air}p_{air}}{V_{L}\;R\;T}*\left(\frac{1-y_{O2}^{\propto }-y_{CO2}^{\propto }}{1-y_{O2}^{\omega }-y_{CO2}^{\omega }}y_{CO2}^{\omega }-y_{CO2}^{\propto }\right)$$ (5)

$$RQ=\frac{CER}{OUR}$$ (6)

Airflow rates Q_air_, gas pressure p_air_ and temperature T were set as constant. The volume of liquid culture medium V_L_ was adjusted for the OUR/CER calculations when feeding was done during the process.

Biomass-specific oxygen uptake rates q_O2_ as well as biomass yield coefficients from oxygen Y_X,O2_ were determined as follows:

$$Y_{X/O_{2}}=\frac{\text{Δ}c_{x}}{\text{Δ}y_{O_{2}}};q_{O_{2}}=\frac{OUR}{c_{x}}=\frac{1}{Y_{X/O_{2}}}\;µ$$ (7)

During the production phase, oil consumption and MEL production rates as well as the MEL yields from oil were calculated according to:

$$r_{oil}=\frac{\text{Δ}c_{oil}}{\text{Δ}t};q_{oil}=\frac{r_{oil}}{c_{x}}=\frac{1}{c_{x}}\;\frac{\text{Δ}c_{oil}}{\text{Δ}t}$$ (8)

$$r_{MEL}=\frac{\text{Δ}c_{MEL}}{\text{Δ}t};q_{MEL}=\frac{r_{MEL}}{c_{x}}=\frac{1}{c_{x}}\frac{\text{Δ}c_{MEL}}{\text{Δ}t}$$ (9)

$$Y_{MEL/oil}=\frac{\text{Δ}c_{MEL}}{\text{Δ}c_{oil}}$$ (10)

Lastly, the percentage of MEL (X_MEL_) in the lipid fraction, which is an indicator for the conversion of the substrate lipids (oil and fatty acids) into product (MEL), was calculated from the measured concentrations of MEL, fatty acids and oil respectively:

$$X_{MEL}=\frac{c_{MEL}}{c_{oil}+c_{FA}+c_{MEL}}=\frac{c_{MEL}}{c_{crude\;extract}}$$ (11)

The value X_MEL_ is also equivalent to the purity of the crude lipid extract, which is obtained when the lipids (MEL, fatty acids and oil) are extracted from the broth as the first step of downstream processing.

Linear regression of experimental data was performed to determine the different rates and yields using professional graphing and analysis software (OriginPro, OriginLab Corporation, United States).

### Kinetic Modeling and Simulation of the Process

For kinetic modeling of the process, several ordinary differential equations (ODEs) were designed and implemented in Microsoft Excel. According to the separation of growth and MEL production phase, two partial models were developed. Numerical solution of the ODEs based on an explicit Euler method with a step size of 0.05 h for the growth model and 0.2 h for the production model was used to simulate the respective concentrations. The model equations and input parameters are described in full detail in the results section.

## Results and Discussion

Based on our previous publication, the MEL fermentation process was generally divided into two stages (Beck and Zibek, 2020a). First, a growth phase was performed on glucose as sole carbon substrate to obtain high cell biomass concentrations, followed by a production phase with rapeseed oil as inductor and main carbon substrate for MEL production. The advantage of such a two-stage process is that both phases can be optimized individually and independently to a certain extent. For example, the effect of biomass concentration on MEL productivity can be studied much better than in a single-stage batch process, where many metabolic pathways are active simultaneously and are competing for the same substrate. According to our previous results, an efficient biomass formation with high cell density is a crucial step for subsequent MEL production. In the current study we therefore optimized first the biomass growth phase by evaluation of medium composition and carbon sources, and developed a batch and a subsequent fed-batch process to increase the biomass concentration in the bioreactor. The amount of oil feeding during production phase was then adapted to the biomass concentration to achieve full substrate conversion. Finally, the experimental data obtained were used to develop a kinetic model that allowed simulation of the process.

### Characterization and Quantification of Cellular Biomass

As a first step, the cell growth of *M. aphidis* was characterized in detail. This is particularly important since *M. aphidis* is known to have a dimorphic growth behavior and to accumulate intracellular storage lipids, which are visible as globules inside the cells (compare Rau et al. (2005b) and Beck and Zibek (2020a)). Both phenomena made it necessary to define how biomass is quantified and how it is composed in order to establish a robust and well-defined fermentation process.

**FIGURE 1:**
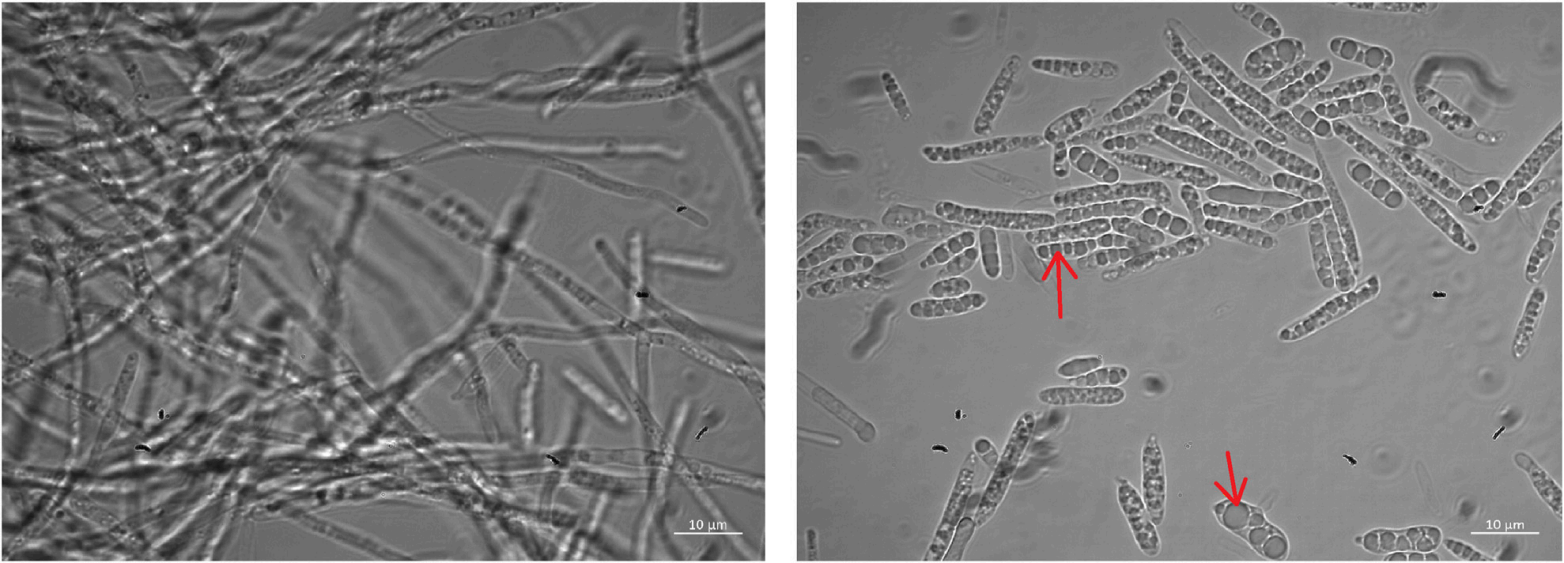
Microscope images of *M. aphidis* cells during growth (left) and MEL production (right). Arrows indicate visible storage globules inside the cells.

Dimorphic growth of *M. aphidis* was observed with our defined mineral medium in the bioreactor, using glucose as the sole carbon source for cell growth (Figure 1). During early growth phase, the cells grow as filamentous cells, which leads to large macroscopic mycelia that are visible to the naked eye. During late growth phase, when the carbon and nitrogen sources are about to be depleted, more and more elongated single cells are visible under the microscope and the broth becomes more homogenous. During production phase on oil, which is running under nitrogen-limited conditions, mostly single cells are present in the culture broth. Moreover, storage vesicles inside the cells are observed during MEL production phase on rapeseed oil, which leads to a swelling of the cells.

As mentioned before, this special behavior of the cells influences the way in which biomass needs to be measured and quantified. We thus evaluated the elemental composition of dried *M. aphidis* cells during the different process stages, i.e. growth and production phase. From a set of more than 20 samples, taken during 12 different fermentations and different process stages in the bioreactor, the average biomass composition during growth and MEL production phase was determined. It was evident that the elementary composition was considerably different between the two phases. While an average nitrogen (N) content of 6.2 ± 1.4% w/w was determined during non-limited growth on glucose and nitrate, this value dropped to only 1.3 ± 0.4% w/w during the production phase on oil (nitrogen-limited). At the same time, relative carbon (C) and hydrogen (H) levels increased (Table 1).

**TABLE 1:** Average biomass composition (CHN-analysis; n > 20 samples from 12 different fermentations) of *M. aphidis* cells during growth phase (glucose, non-limited) and MEL production phase (oil addition, N-limited).

Phase and Respective Substrate	(g/100 g_biomass_)				Molar Composition
	C	H	N	Others (O, S, Minerals)	
Growth (glucose)	48.5 ± 3.9	7.1 ± 0.6	6.2 ± 1.4	38.1 ± 3.7	CH_1.77_N_0.112_O_0.597_
Production (oil)	62.2 ± 3.0	9.2 ± 0.5	1.3 ± 0.4	27.3 ± 3.3	CH_1.77_N_0.018_O_0.333_

In order to set up a balance of elements, the relative C, H and N contents were multiplied with the measured dry biomass at the respective time points, yielding the total amount of each element contained within the cellular biomass. It was found that the observed increase of measured dry biomass during production phase was caused only by accumulation of C and H, while the total mass of elementary nitrogen in the dry cell mass remained constant after the depletion of NaNO_3_ in the medium. In our culture medium using 3 g/L NaNO_3_ as nitrogen source, a maximum of 0.49 g_N_/L elementary nitrogen was available. At an average nitrogen content of 6.2 ± 1.4% in the biomass, the medium would thus allow for a theoretical maximum biomass concentration of 7.9 ± 1.9 g/L during growth. The theoretically maximum biomass yield from sodium nitrate Y_X/NaNO3,max_ is thus equal to 2.6 g/g. During our experiments, an average biomass yield from nitrate Y_X/NaNO3_ of 1.8 g/g (i.e. around 5.5 g/L biomass from 3 g/L NaNO_3_) was observed during growth. The nitrogen yields were hence at 70% of the theoretical maximum. The further strong increase of biomass during production phase, which was observed up to around 30 g/L dry mass, was therefore only caused by C and H accumulation (lipid inclusion) and not by actual “growth” in terms of cell doubling, which would require additional nitrogen for DNA, RNA and amino acids synthesis.

Similar observations were made by Rau et al. (2005b), who already showed that intracellular protein levels remained constant after initial growth although dry biomass further increased. This was reported to be due to accumulation of lipid material inside the cells. An elemental composition of C 68.1, H 8.0 and N 1.9 (% w/w) was presented for *M. aphidis* DSM70725 biomass during simultaneous growth and MEL production on soybean oil (Rau et al., 2005b), which is similar to our values during production phase on oil. Moreover, Klement et al. (2012) have also shown a strong decrease in cellular nitrogen content between exponential growth and nitrogen limiting conditions for the closely related *Ustilago maydis* (N 7.35 vs 2.83% w/w) during itaconic acid production. Both references confirm our observations of a decreasing cellular nitrogen content during the nitrogen-limited MEL production phase on oil.

It was hence concluded that using lipid substrates does not only result in biomass increase by cell division but mostly due to simultaneous accumulation of lipid storage material inside the cells. This is one of the major reasons why we decided to work with separate growth and MEL production phases, allowing for a more precise analysis of biomass concentration during the fermentation process. Whenever biomass-specific rates were calculated, they were based on the biomass concentration that was achieved at the end of the growth phase (c_x,growth_). It also needs to be mentioned that the lipid inclusion has implications for the modeling and simulation of biomass and lipid concentrations during the production phase, as some of the lipid substrate is accumulated in the biomass and is thus not available for MEL production.

### Evaluation of Medium Composition with Regard to Biomass Formation and MEL Production

While most of the elementary nitrogen supplied in the culture medium was actually recovered in the produced biomass at the end of the growth phase, as demonstrated before, the elementary carbon was less efficiently directed into biomass formation. Of the initial 12 g_C_/L elementary carbon in our medium containing 30 g/L glucose, only around 2.65 g_C_/L (i.e. 22 % of the initial amount) were found in the biomass at the end of the batch growth phase. This led to a biomass yield coefficient from glucose (Y_X/glucose_) of 0.18 g/g. Even when taking into account that CO_2_ formation during cellular respiration can amount for up to 50% of carbon consumption in aerobic processes, there is still a considerable amount of elementary carbon not directed into either biomass formation or cellular respiration.

It was expected that increasing the concentration of the most important macro elements N, P or S in the culture medium could lead to a higher biomass yield from the same amount of glucose and thus a higher biomass concentration. Hence, a possible adjustment of our mineral medium was investigated. Different medium compositions with increased concentrations of N, P and S were examined at a fixed glucose concentration (30 g/L), resulting in lower C/N, C/P and C/S ratios. The experimental design was based on a randomized Box-Behnken design (Supplementary Table S1).

Statistical analysis of the results by ANOVA showed that nitrogen was the most significant factor (*p* < 0.0001) for biomass formation, which was quantified as dry biomass concentration at the end of the growth phase (48 h). Phosphate was less significant (*p* = 0.033), while sulfur was not significant (*p* = 0.526) and was thus excluded from the final biomass model. Biomass formation was best described by a reduced quadratic model using N^2^, N and P as model terms. Comparison of experimental biomass values with those predicted by the quadratic model showed very good agreement. The two verification runs, placed at the corners of the design space, were also in agreement with the model prediction. For the MEL production, which was quantified as the MEL concentration after 210 h, only the nitrogen concentration was a significant factor (*p* = 0.0002). A reduced linear model using only N as significant parameter was the best fit for the data. Similar as for the growth model, comparison of experimental and predicted values as well as the two verification runs showed good agreement.

**FIGURE 2:**
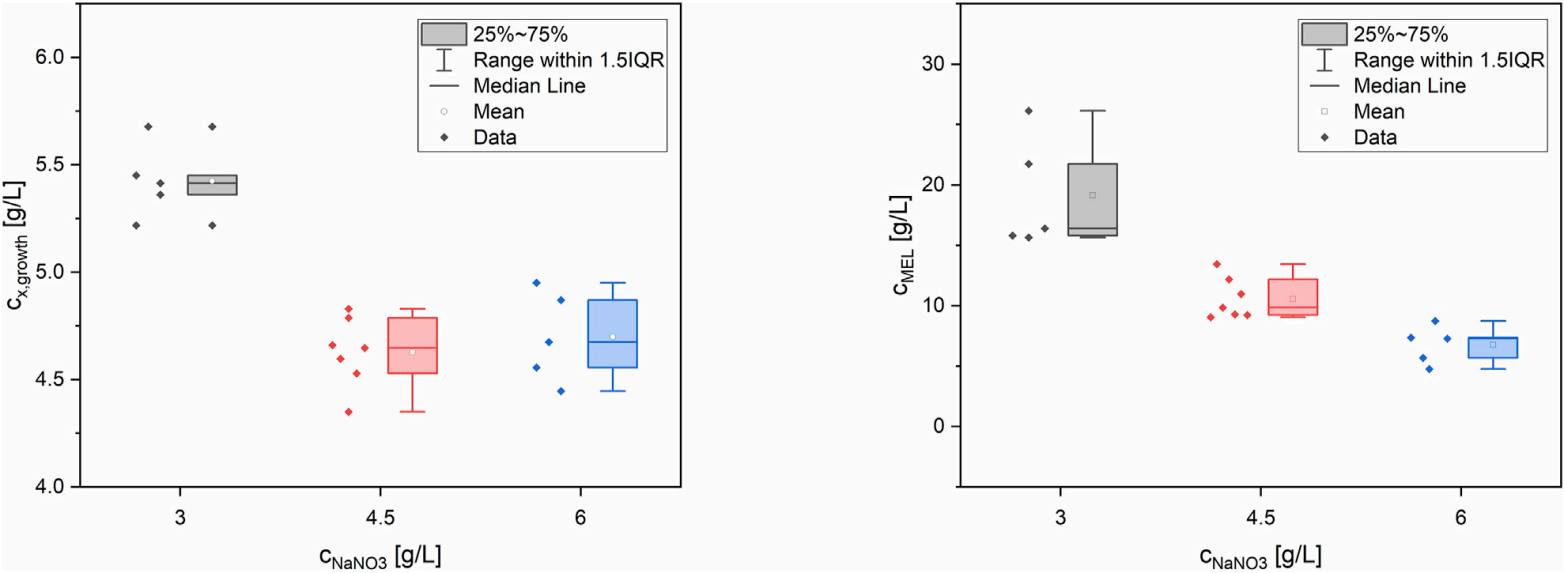
Box-whisker plots for biomass (left) and MEL concentration (right) depending on NaNO_3_ concentration in the medium, at a fixed glucose concentration of 30 g/L. The different concentrations of P and S were less or even not significant.

**FIGURE 3:**
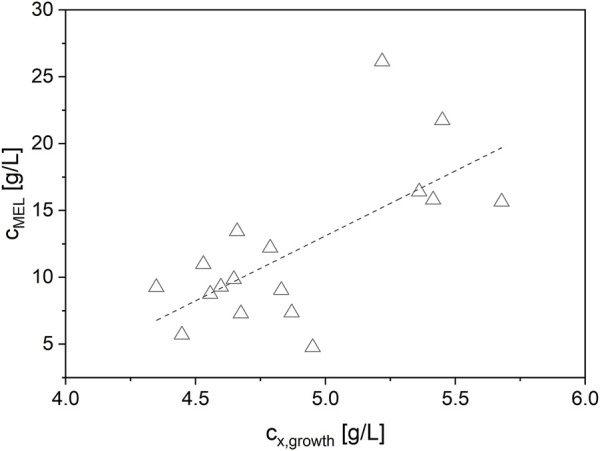
Positive correlation between the target values biomass and MEL concentration. Higher biomass concentrations at the end of the growth phase lead to higher MEL concentrations during production phase.

In summary, the lowest concentration of NaNO_3_ (3 g/L) and thus the highest C/N ratio of 28.4 mol_C_/mol_N_ yielded the best biomass formation (c_x,growth_ = 5.4 ± 0.2 g/L) from glucose and subsequently the highest MEL production (c_MEL_ = 19.1 ± 4.6 g/L) from rapeseed oil (see Figure 2). The maximum biomass yield coefficient from glucose Y_X/glucose_ was 0.18 ± 0.01 g/L. In terms of C/P ratio, a higher P concentration slightly increased biomass but at the same time did not have a significant influence on MEL formation. The effect of P concentration was overall very small compared to N. Moreover, a positive correlation between the target values biomass and MEL concentration was observed (Figure 3). MEL formation is enhanced when higher amounts of biomass are formed during growth phase. This is in agreement with the theory of secondary product formation, e.g. by Luedeking and Piret (1959), and similar to observations made by Kitamoto et al. (1992) using a resting cell approach with different cell concentrations. It also confirmed our prior observations using this mineral medium at different starting concentrations, where higher biomass concentrations led to subsequently higher MEL production from oil in several Ustilaginaceae species (Beck and Zibek, 2020a).

Contrary to our initial expectation, it was concluded that high C/N, C/P and C/S ratios in the medium are best to form high biomass and MEL concentrations with *M. aphidis*. Specifically, the high C/N ratio in our mineral medium with 30 g/L glucose and 3 g/L NaNO_3_ proved to be ideal for biomass formation and subsequent MEL production. Most likely the resulting nitrogen limitation is beneficial for induction of MEL production as a part of the secondary lipid metabolism, which is promoted under such conditions (Hewald et al., 2006; Bolker et al., 2008; Beck and Zibek, 2020b). This holds also true for other fungal glycolipid biosurfactants like sophorolipids or cellobiose lipids (Jezierska et al., 2018). Moreover, it has been reported before that an ideal C/N ratio for the growth of *M. aphidis* DSM 70725 was around 42.5 mol_C_/mol_N_ (30 g/L glucose and 1 g/L NH_4_NO_3_) and that a variation of the phosphate content between 0.1 g/L and 0.9 g/L KH_2_PO_4_ did not have an influence neither on cell growth nor on MEL production (Rau et al., 2005b). In Rau et al. (2005b), the C/N ratio was even higher than in our work, but it should also be noted that yeast extract was used in their culture medium, which contains additional carbon, nitrogen and phosphate compounds and thus influences these ratios to a certain extent.

According to the presented results, it was decided to continue with the mineral medium composition containing 30 g/L glucose, 3 g/L NaNO_3_, 1 g/L KH_2_PO_4_ and 1 g/L MgSO_4_*7H_2_O for all further experiments.

### Screening of Different Sugar Substrates for Growth

After optimizing the mineral medium composition for biomass formation, different carbon substrates for cell growth were screened as well. The investigated sugars were glucose, sucrose, fructose, xylose, arabinose and cellobiose. Moreover, several fractions from the sugar refining process like syrup, two different process water A (containing mostly sucrose) and B (containing fructose and glucose) from process centrifuges, as well as sugar cane and sugar beet molasses were evaluated. Glucose was used as the reference sugar for growth. All carbon substrates were employed at the same total sugar concentration of 30 g/L.

**FIGURE 4:**
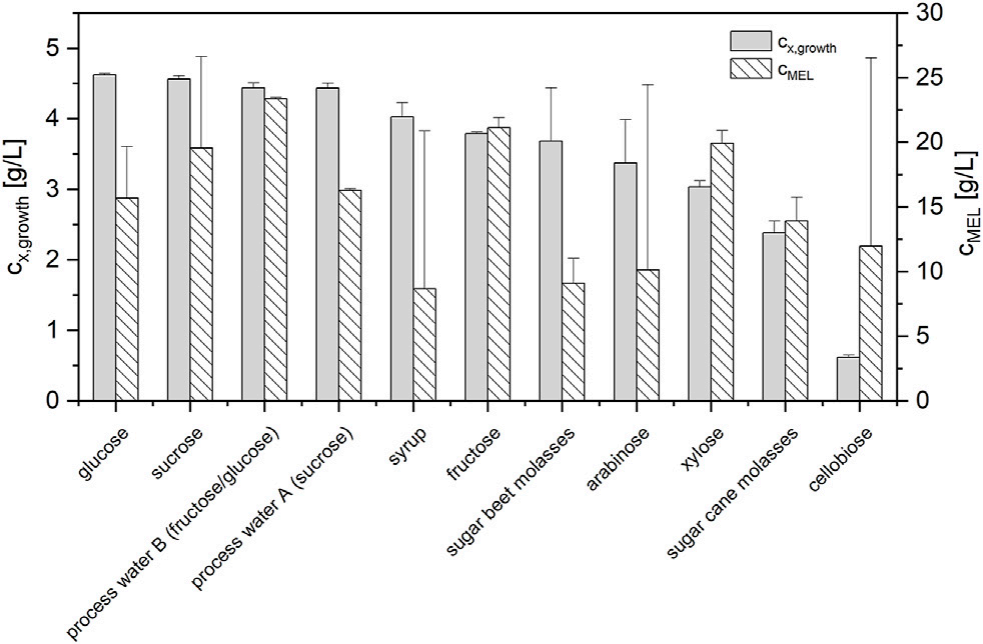
Maximum biomass concentrations (*n* = 3) for different sugar substrates during growth and corresponding MEL concentrations (*n* = 2) after a subsequent production phase on 8% rapeseed oil. All sugar substrates were employed at a total sugar concentration of 30 g/L to ensure direct comparability.

The results showed that the crystalline sugars sucrose, fructose, xylose, and arabinose were fully consumed at the end of the growth phase and yielded biomass concentrations that were in the same range as those from the glucose reference (3.0–4.6 g/L, see Figure 4; Supplementary Table S2). The different fractions from sugar refining, which contained mostly sucrose, glucose and fructose, were also fully assimilated for growth and yielded similar biomass concentrations. Of all the investigated substrates, only cellobiose was shown to be unsuitable for cell growth. Cellobiose was not assimilated by the microorganism and resulted in much lower biomass concentrations (0.62 g/L).

In the subsequent production phase on rapeseed oil, MEL was formed in all experiments (Figure 4). Thus, none of the sugars inhibited subsequent MEL formation. The reference process with glucose yielded an average MEL concentration c_MEL_ of 15.7 ± 4.0 g/L. Compared to the reference, sucrose, fructose and xylose as well as process water B had even higher average c_MEL_. Process water A was similar to glucose in terms of c_MEL_. Syrup, arabinose and cellobiose as well as the two molasses had lower average c_MEL_. However, there was also a high standard deviation for syrup, arabinose and cellobiose, as one experiment had a c_MEL_ comparable to the glucose reference, while the duplicate did not produce MEL. The successful production of MEL in the experiments with cellobiose, despite the absent cell growth during the growth phase, could be attributed to a combined cell growth and MEL production from rapeseed oil.

Overall, it was demonstrated that *M. aphidis* is able to assimilate a wide range of sugars for growth and that these sugars did not negatively influence or inhibit subsequent MEL formation. This is the first time that such a screening has been reported for *M. aphidis*. Nevertheless, it had been shown before that the closely related fungus *M. antarcticus* is able to use glucose, sucrose, fructose, mannose, mannitol and glycerol as carbons sources for growth, and that neither of the sugars negatively influenced subsequent MEL production from soybean oil (Kitamoto et al., 1992). Thus, it can be expected that most of the related Ustilaginaceae species that are employed for MEL production have the ability to use these carbon substrates for cell growth.

It was then decided to continue with glucose as growth substrate for further experiments, despite the equally good results for many of the investigated sugars. Glucose had been used before in other publications, which ensures comparability, and it was now shown to yield high biomass and subsequently high MEL concentrations in a reproducible manner with *M. aphidis* as the production strain.

### Bioreactor Fermentations With Batch Growth Phase

The fermentation process for MEL production was then transferred to a 7-L aerated stirred-tank bioreactor and different process variations were investigated. Initially, a process with batch growth phase was established that served as a benchmark for further process optimization.

**FIGURE 5:**
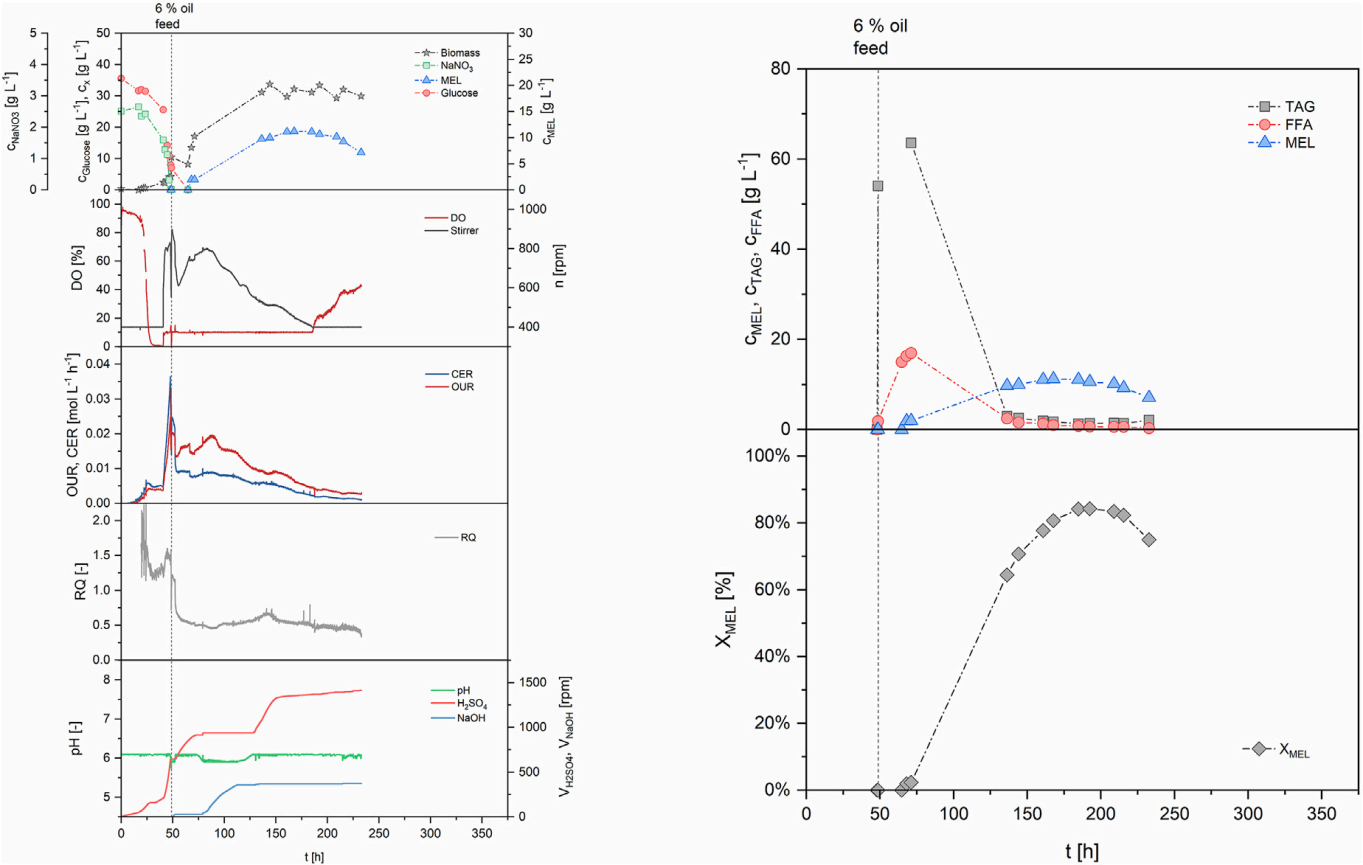
Process data for the two-staged batch (B1). Growth was performed in batch mode, and production was initiated at 48 h using 6% v/v rapeseed oil as substrate.

The first run in the STBR was based on a simple two-staged batch (B1, see Table 2). Batch growth was performed on our defined mineral medium with 30 g/L glucose as carbon substrate and production was initiated with 6% v/v rapeseed oil after 48 h, when the cells entered stationary phase (Figure 5). The consumption of primary growth substrates glucose and nitrate resulted in formation of 4.2 g/L biomass, measured as cell dry weight. Parallel to cell growth, oxygen demand of the cells increased exponentially during growth, showing a maximum OUR of 24.9 mol/Lh after 48 h. Maximum CER at the same time was at 36.5 mmol/Lh, and the average RQ during growth phase was 1.39. The biomass specific rates q_O2_ and q_CO2_ were 4.2 and 6.5 mmol/gh respectively. Dissolved oxygen concentration in the medium decreased until the 10% set point was reached, where it was then maintained by increasing stirring speed. The growth-associated increase of pH, which resulted from a consumption of substrates, was stabilized by automated addition of acid to maintain pH 6 during growth. At 48 h, when the nitrogen source was completely consumed, rapeseed oil (6% v/v, reference volume of 4 L) was added to start the MEL production phase. Oil addition negatively influenced the offline measurement of OD and biomass during the first hours after oil addition due to the formation of a separate phase, but was again showing reasonable values during later stages when oil had been (partly) hydrolyzed. Oil hydrolysis by extracellular lipases began directly after the oil (triacylglyceride, TAG) was added to the fermenter, resulting first in an increase of free fatty acids (FFA) in the broth and then MEL production. The measured biomass concentration ultimately increased up to around 30 g/L during production phase, due to an intracellular accumulation of lipids as discussed before. A plateau of MEL concentration was reached when nearly all fatty acids were consumed after 150–170 h. At 168 h, a MEL concentration of 11.2 g/L was obtained. The share of MEL in the crude extract (X_MEL_), which was calculated as the percentage of MEL (i.e. 11.2 g/L) in the crude lipid extract (13.9 g/L), was 81% at this time. The rest was remaining substrate oil (12%) and free fatty acids (7%). The average MEL formation rate (r_MEL_) between 48 and 168 h was calculated to 0.105 g/Lh and the yield coefficient with regard to the total amount of oil added was calculated to 0.226 g/g_oil_. Despite the almost full consumption of oil and fatty acids, the MEL yield coefficient was relatively low. This can be explained by the previously described accumulation of storage lipids inside the cells, which leads to an increase in cell biomass, but makes them unavailable for MEL production. The process was maintained until 240 h, but there was even a decrease in MEL concentration after that point, correlating with a starvation of the microorganisms. Off-gas analysis during the production phase showed a decrease of cellular respiration, with OUR and CER values that were lower than during exponential growth and constantly decreasing with time. During the production phase the RQ was around 0.52. Hence, the oxygen uptake was higher than the emission of CO_2_, which indicates that a highly oxidative pathway was active. This is for example the case during lipid metabolism, where the fatty acids are converted to acetyl-CoA by multiple cycles of β-oxidation to generate energy. The trend of pH was also an interesting indicator. As long as the oil was hydrolyzed, which correlated with a decrease in oil concentration and a release of fatty acids, the pH decreased, so base was constantly titrated to maintain the pH. At the time when oil and also fatty acid concentrations reached zero (around 130 h), pH of the broth shifted and acid was titrated. This was accompanied by a decrease in oxygen uptake and stirring speed, which can be attributed to the depletion of substrate and thus lower respiratory activity. This behavior had been observed before in the microcultivation experiments with our mineral medium, where the dissolved oxygen and pH in the broth showed the same trends (see also Beck and Zibek (2020a)). Therefore, the recorded online values (DO, stirring speed, OUR and pH) could be used to help interpret the time course of biomass growth, oil hydrolysis and MEL production.

**TABLE 2:** Process values for fermentations with batch growth and single or repeated oil feeding.

Process	B1	B2
growth phase	batch	batch
c_x,growth_ (g/L)	4.2	n.d
Y_X/gluc_ (g/g)	0.155	n.d
OUR_max_ (mmol/Lh)	24.9	25.9
production phase	single oil feed	repeated oil feed
total oil feed (%)	6	22 (6 + 4 × 4)
oil-to-biomass ratio (g/g)	12.0	n.d
c_MEL,max_ (g/L)	11.2	27.1
r_MEL,av_ (g/Lh)	0.105	0.101
MEL yield per total oil^#^ (g/g)	0.226	0.135
X_MEL_ (%)	81	20
process duration (h)	170*/240	333

^#^ related to the total amount of oil added to the reactor.

* time when maximum MEL, concentration and/or purity were reached.

Overall, the values for biomass and MEL concentration in this first bioreactor experiment were comparable to those obtained in the microcultivation system before. As the experiments in the microcultivation system were also performed as a two-stage batch process, the process was successfully transferred from a shake culture to the stirred-tank system.

In a second bioreactor experiment (B2, Table 2; Supplementary Figure S1), production phase was initiated again with 6% v/v oil after an initial batch growth phase, but was then prolonged by multiple additions of rapeseed oil in portions of 4% v/v after 118, 165, 215 and 286 h. Maximum oxygen uptake rate during batch growth was at 25.9 mmol/Lh and thus almost identical to the previous run, showing good reproducibility of the batch growth phase. The same was true for CER and RQ. During production phase, a maximum of 27.1 g/L MEL was achieved after 333 h, with an average MEL formation rate of 0.101 g/Lh. The MEL formation rate was thus almost identical to before, while the total MEL concentration was increased due to the longer process time and higher substrate concentration. However, the share of MEL (X_MEL_) in the crude lipid extract at the end of the process (132.6 g/L crude extract) was only at 20%, meaning that a high amount of oil and fatty acids (32 and 48% respectively) remained in the broth. This was due to the multiple oil feeds and thus higher concentrations of fatty acids released, which could not be consumed by the microorganism and were thus accumulating in the broth over the entire production phase. Oxygen uptake rates and stirring during production phase were maintained at a higher level than before and did not show such a strong decrease, which was indicative for the maintenance of cellular activity by the still active lipid metabolism. Besides, a steady consumption of NaOH was observed to maintain the pH until the end of the process, and no acid was titrated. This correlated with the ongoing oil hydrolysis and accumulation of fatty acids in the broth.

Overall, a higher MEL concentration was achieved in the second process with repeated oil feeding, but only at the expense of an increased process time as well as higher concentrations of remaining oil and fatty acids, which in turn resulted in a reduced purity of the crude extract. Hence, the amount of oil feeding in the second process was too high for the microorganism to convert into product. MEL formation rate was similar between the two processes, regardless of the amount of oil added. This was a clear indication that the MEL formation rate mainly depends on the biomass concentration. Therefore, the biomass concentration should be increased to achieve higher productivity throughout the process. In addition, the oil dosage needs to be adjusted to the current biomass concentration to achieve good oil conversion, which in turn should lead to lower fatty acid concentrations and thus higher purity of the crude lipid extract at the end of the process.

### Bioreactor Fermentations With Increased Biomass Concentration Using Fed-Batch Growth

Based on the previous results, higher biomass concentrations during growth phase were then targeted, which were expected to result in higher MEL formation rates and better oil conversion during production phase. In a first approach, the initial batch concentration of all medium components was increased to levels of 60, 90 or 120 g/L glucose respectively and the growth was examined in the microcultivation system and STBR (data not shown). Although the growth substrates (glucose and NaNO_3_) were fully consumed and higher biomass concentrations were formed at the end of the growth phase, negative effects like oxygen limitation, growth inhibition and biomass accumulation at the reactor vessel occurred at these high medium concentrations in the batch, which in turn led to lower reproducibility and stability of the process. Similar to our observations, it had been reported before that batch concentrations of glucose and NaNO_3_ above 30 and 3 g/L, respectively, did not lead to higher biomass concentrations and subsequently even lower MEL yields (Rau et al., 2005b).

Consequently, we focused on developing fed-batch processes with exponential substrate feeding during the growth phase. Exponential feeding ensures microbial growth at a defined and constant specific growth rate, leading to high biomass formation in a reproducible manner, while at the same time maintaining low concentrations of medium components in the reactor. The exponential feeding phase was always initiated after an initial batch growth phase, which was performed identically to the previous batch processes to ensure reproducibility and comparability. The aim of all fed-batch processes was to obtain a biomass concentration that was at least twice that of a simple batch growth phase, and subsequently to prove that this led to an increased MEL production rate during production phase.

**FIGURE 6:**
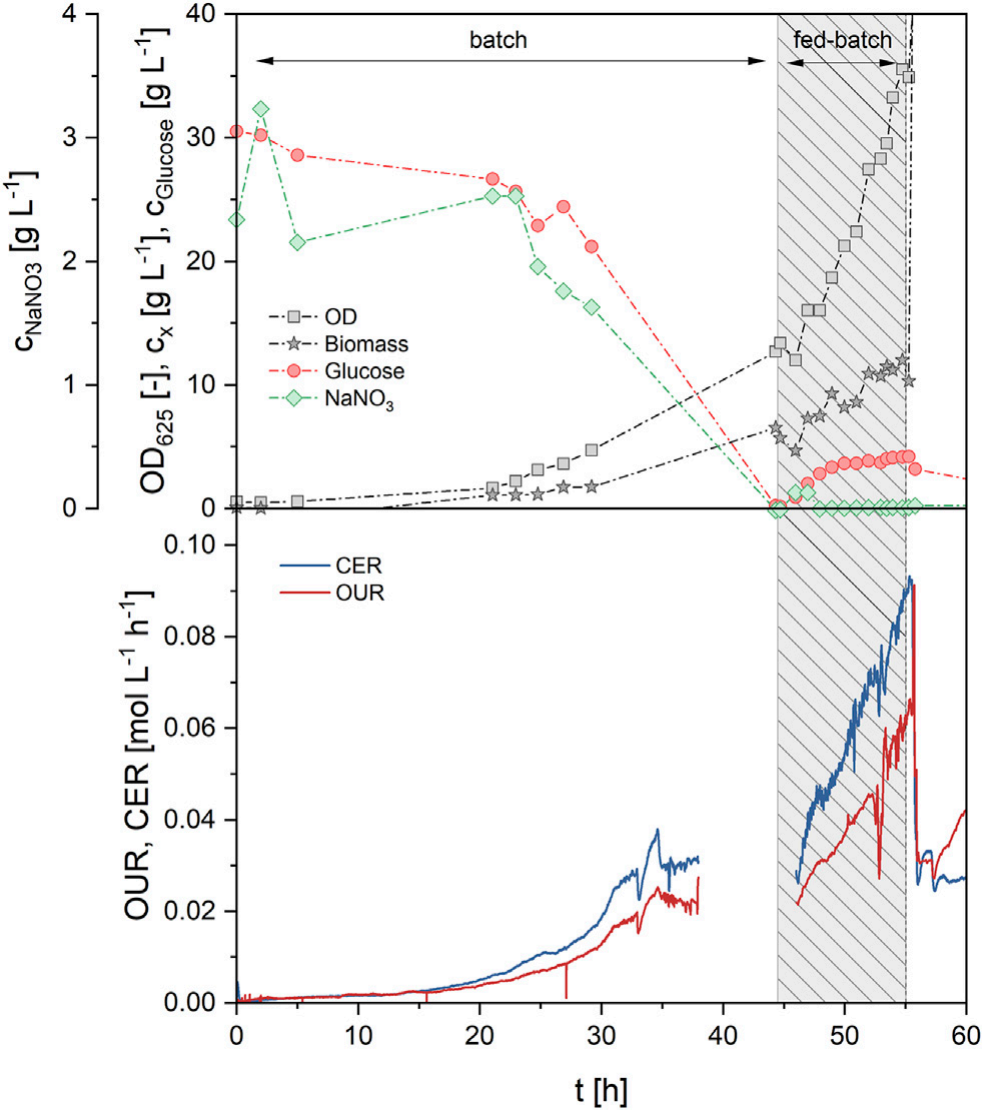
Process data of fed-batch process 1 (FB1) during growth phase (0–55 h) showing concentrations of glucose, NaNO_3_, biomass and OD (upper) and off-gas analysis (lower graph). Medium feeding was applied from 44.5–55 h with an exponential feed rate at µ_set_ = 0.08 h^−1^, leading to a further increase of biomass, OUR and CER.

In the first fed-batch process (FB1, Table 3; Supplementary Figure S2), exponential feeding with a growth rate µ_set_ of 0.08 h^−1^ was carried out after an initial batch growth phase on 30 g/L glucose. The growth rate for fed-batch was thus set at roughly 70% of the maximum growth rate µ_max_, which was determined as 0.11 h^−1^ in the batch growth experiments before. Other parameters for the feeding equation were set at c_x0_ = 7.6 g/L and Y_X/S_ = 0.25 g/g. Exponential feeding was conducted for 10.5 h between 44.5 and 55 h of process time. During that time, a total of 460 g of feeding solution were added, equivalent to 138 g glucose and 13.8 g NaNO_3_. This led to a biomass increase from 5.7 g/L (at the end of batch growth) to 12.0 g/L (end of fed-batch), thus more than doubling the biomass concentration. The corresponding biomass yield from glucose was determined as 0.171 g/g and was constant between batch and fed-batch phase. Parallel to the biomass concentration, the OUR increased from 25.0 mmol/Lh during batch growth up to 58.2 mmol/Lh at the end of the fed-batch phase, showing the proportionality between biomass concentration and OUR (Figure 6). CER was simultaneously increased from 31.6 mmol/Lh up to 82.2 mmol/Lh. The specific rates q_O2_ and q_CO2_ during the fed-batch phase were at 4.3 and 6.5 mmol/gh, respectively, and the average RQ was 1.42 during both batch and fed-batch growth. Generally, off-gas data was identical with the results from batch fermentations, showing the high reproducibility and robustness of the biomass growth phase. While nitrate concentration in the broth remained at limiting levels during the feeding phase, a slight glucose accumulation up to 4.2 g/L was registered. Production phase was initiated at 55 h by adding 6% v/v rapeseed oil to the bioreactor. The first oil feed was converted very fast, showing a MEL concentration of 18.5 g/L at a X_MEL_ of 73% after 142 h. Three further oil feeds of 6% v/v each were done at 142, 190 and 242 h to maintain MEL production phase. Subsequently, a MEL concentration of 43 g/L was reached after 310 h, but X_MEL_ was only at 40% at that time. The process was thus continued up to a process time of 500 h without further oil feeding. MEL concentration increased only a bit and reached a final value of 50.5 g/L, but the X_MEL_ was increased to 68% again, due to further consumption of remaining oil and fatty acids (8 and 24% at the end respectively). The average MEL production rate between 55 and 310 h was relatively constant at 0.185 g/Lh, and therefore almost doubled in comparison to the previous batch processes. After 310 h, the production rate decreased, which was probably caused by the long process time and reduced activity of the cells.

**TABLE 3:** Process values for fermentations with combined batch and fed-batch growth and repeated or continuous oil feeding.

Process	FB1	FB2	FB3	FB4
growth phase	batch + fed-batch	batch + fed-batch	batch + fed-batch	batch + fed-batch
µ_set_ (h^−1^)	0.08	0.09	0.09	0.08
feeding duration (h)	10.5	11.5	11	16.9
total medium feed (g)	460	612	708	701
c_x,growth_ (g/L)	12.0	15.5	10.9	14.1
Y_X/gluc_ (g/g)	0.171	0.212	0.171	0.254
OUR_max_ (mmol/Lh)	58.2	74.8	58.0	63.2
production phase	repeated oil feed	continuous oil feed	repeated oil feed	continuous oil feed
total oil feed (% v/v)	24 (6 + 6 + 6 + 6)	24 (6 + 18 cont.)	12 (6 + 6)	12 (6 + 6 cont.)
oil-to-biomass ratio (g/g)	20.2	15.7	10.9	8.8
c_MEL,max_ (g/L)	50.5	43.9	35.7	34.3
r_MEL,av_ (g/Lh)	0.185	0.422	0.335	0.378
MEL yield per total oil^#^ (g/g)	0.208	0.181	0.294	0.275
X_MEL_ (%)	68	49	88	98
process duration (h)	502	147*/310	170*/231	169*/307

^#^ related to the total amount of oil added to the reactor.

* time when maximum MEL, concentration and/or purity were reached.

The second fed-batch process (FB2, Table 3; Supplementary Figure S3) was conducted at a slightly higher exponential feeding rate, equivalent to a µ_set_ of 0.09 h^−1^. In total, 612 g of feed solution (184 g glucose and 18.4 g NaNO_3_) were added during an 11.5 h feeding period between 42.5 and 54 h. This resulted in an increased biomass concentration of 15.5 g/L at the end of the fed-batch. The maximum OUR by that time was 74.8 mmol/Lh. Biomass concentration and OUR were therefore further increased compared to the previous run. Production phase was initiated with 6% v/v rapeseed oil at 54 h as previously, but this time the additional 18% v/v of oil were delivered as a continuous feed with a constant rate of 11 ml/h between 72 and 140 h. Compared with the previous process, the same amount of oil was added in a shorter time period. This led to a faster oil hydrolysis and high fatty acid (67 g/L) and MEL concentrations (43.9 g/L) after 147 h, when the continuous oil feeding was stopped. The average MEL formation rate between 54 and 147 h was calculated to 0.422 g/Lh. Due to the still high fatty acid concentration, however, the share of MEL in the lipid fraction X_MEL_ was only 33% at that time (15% oil and 51% fatty acids respectively). Until the end of the process, fatty acid concentration then decreased again, but MEL concentration was also slightly decreased. Overall, a final X_MEL_ of 49% (11% oil and 40% fatty acids) was obtained after 310 h of process.

Both processes with combined batch and fed-batch growth phase, FB1 and FB2, successfully achieved the goal of higher MEL concentrations and higher MEL formation rates. Compared to the batch process B2, which had a MEL concentration of 27.1 g/L, the two fed-batch processes FB1 and FB2 led to increased MEL concentrations (50.5 and 43.9 g/L). At the same time, MEL formation rate was also increased. This confirmed our hypothesis that a higher biomass concentration at the end of the growth phase leads to faster MEL production. Nevertheless, the share of MEL X_MEL_ in the lipid fraction at the end of the processes was still relatively low, due to remaining oil and fatty acids in the broth. Oil feeding with 24% v/v or around 16–20 g_oil_/g_biomass_ was thus too high, even for the increased cell biomass concentrations. It was therefore decided to decrease the amount of oil feeding to 12% v/v or around 10 g_oil_/g_biomass_, respectively, and to aim for a full conversion of the substrate oil and fatty acids into MEL. Based on an average oil hydrolysis rate of ∼1 g/Lh in the process FB1, this was expected to result in full oil hydrolysis at around 150 h and subsequently a consumption of remaining fatty acids afterwards. The idea was to obtain a X_MEL_ of >80%, i.e. less than 20% remaining oil and fatty acids, similar to the first batch process with single oil feeding (B1).

The third fed-batch process (FB3, Table 3; Supplementary Figure S4) was conducted with the same feeding rate as before (µ_set_ = 0.09 h^−1^) during growth phase (40–51 h). During 11 h of feeding, a total of 708 g feeding solution were added. This resulted in 10.9 g/L biomass and a maximum OUR of 58.0 mmol/Lh. Despite the same feeding strategy as previously, a lower biomass concentration was obtained, which also resulted in an accumulation of glucose and nitrate in the medium. By analyzing off-gas values it was observed that the transition between batch and fed-batch was initiated too late, leading to a partial lysis of cells and a decrease in OUR towards the end of the batch growth phase. Hence, the feeding rate, which was calculated based on an active biomass concentration of 6 g/L at the beginning of feeding, was over-estimated, leading to an accumulation of glucose and nitrate over the fed-batch phase. Production phase was initiated by addition of 6% v/v oil at 51 h and maintained by another 6% at 72 h. Fast oil hydrolysis and almost full consumption of residual oil and FA after 150–170 h were observed, which correlated also with the pH trend as described before. MEL concentration and X_MEL_ reached a plateau at around 170 h. After that time, a decrease in OUR and a slight decrease in biomass were observed until the process was ended at 231 h. At the end, a MEL concentration of 35.7 g/L and a X_MEL_ of 88% in the crude extract (12% fatty acids and 0% oil) were measured. Average MEL formation rate between 51 and 170 h was at 0.335 g/Lh. The MEL yield coefficient with regard to the amount of oil added was 0.294 g/g, and therefore increased from the previous processes. Although the process was ultimately terminated after 231 h, it could have been stopped already at around 170 h, where MEL concentration and X_MEL_ were at their maximum (36.8 g/L and 92% respectively). Overall, the goal of full oil conversion into MEL, while at the same time achieving a high MEL concentration and productivity, was achieved in this process.

The fourth and final fed-batch process (FB4, Table 3; Supplementary Figure S5) was combining the results from previous runs. The initial batch concentration of all medium components was lowered to an equivalent of 20 g/L glucose, respectively, and the fed-batch phase with µ_set_ = 0.08 h^−1^ was started earlier to prevent starving of cells between batch and fed-batch phase. By adding 701 g of feeding solution between 27 and 44 h of process time, a maximum biomass concentration of 14.1 g/L was reached. This time the transition between batch and fed-batch was very smooth and showed a continuous and exponential increase of OUR throughout the growth phase up to a maximum OUR of 63.2 mmol/Lh. Nitrate was fully consumed at the end of the fed-batch, while the glucose concentration remained at 12 g/L. Production phase was then initiated at 44.5 h by adding 6% of oil to the fermenter. Further oil feeding (6% v/v) was done continuously at a rate of 8 ml/h between 52 and 82 h. The total amount of oil addition was therefore 12% v/v as before. After 169 h, a maximum MEL concentration of 34.3 g/L was obtained. Average MEL formation rate was 0.378 g/Lh (45–140 h) and thus similar to FB3. The calculated yield coefficient with regard to the total amount of oil added was at 0.275 g/g. Due to remaining oil and fatty acids, the X_MEL_ at that time was at 69%. Therefore, the process was prolonged to 307 h and the purity increased to 98% as almost all lipids were consumed (only 1% fatty acids and 1% oil remaining). MEL concentration was slightly decreased to 30.0 g/L, but remained more or less at the same level.

Overall, the last two fed-batch processes FB3 and FB4 were very comparable in terms of MEL concentrations, formation rates and yields, and they were both showing low residual oil and fatty acid concentrations at the end of the process. Both processes hence achieved a X_MEL_ > 90% in 170–200 h of process time. This was similar to the first batch process B1, but at an increased MEL concentration of around 35.7 or 34.3 g/L (FB3 and FB4) instead of 11.2 g/L (B1). The ratio of oil addition with regard to biomass concentration in those processes was in the range of 9–11 g_oil_/g_biomass_ resulting in almost full substrate conversion. When this ratio was higher, like in B2, FB1 or FB2, a higher residual amount of fatty acids and thus lower purity of the crude lipid extract at the end of the process was obtained. A ratio of around 10 g_oil_/g_biomass_ can therefore be seen as an ideal value for determining the amount of oil feeding. Although not stated explicitly, the amount of oil with regard to biomass in the publication of Rau et al. (2005a) was estimated to ∼10 g/g as well, taking into account their values for cell protein concentration and overall oil feeding. This also resulted in full conversion of oil and fatty acids after 288 h (Rau et al., 2005a) and is thus in good agreement with our results.

### General Observations During MEL Production in Bioreactors

Several general observations were made in all of the processes presented. First, foaming occurred towards the end of the batch growth phase and further increased during fed-batch phase when higher biomass concentrations were reached. Starting from very coarse and dry foam during early growth, the diameter of the foam bubbles decreased to only a few mm over time and became increasingly wet. It is generally hard to determine which effect is dominating the foam formation during growth phase. Parallel to the increasing concentration of cells, the stirrer speed was automatically increased to enhance the oxygen transfer, which influences the hydrodynamics and thus foam formation in the bioreactor. Moreover, medium components like nitrate are limiting towards the end of the batch and during the fed-batch growth phase, which could also have an influence on foam formation. Ultimately, the mechanical foam destruction, which had been installed in the headspace of the reactor, was no longer effective after a certain point, leading to a partial overflow of culture broth into the exhaust air tubing. Nevertheless, the addition of rapeseed oil to start the production phase caused the foam to be destroyed again. Oil could thus act not only as substrate, but also as a chemical antifoaming agent (see also Rau et al. (2005a)). During production phase, foaming was less problematic and the process remained stable. Overall, it was concluded that a smooth transition from (fed-batch) growth phase to production phase is crucial for achieving a stable process without foam overflow.

Several online-recorded values proved to be good indicators for process control and monitoring. Oxygen uptake and carbon dioxide emission rates, which were obtained from off-gas analysis, correlated with biomass growth and substrate consumption during growth phase. The biomass specific rates q_O2_ and q_CO2_ were 4.3 and 6.5 mmol/gh, respectively, and remained constant during batch and fed-batch growth. This is the first time that a biomass specific oxygen demand has been determined for a producer strain in the MEL production process. Off-gas analysis even proved a better indicator for cell growth than the off-line measurement of biomass, as it was not affected by foaming or inhomogeneity in the broth. Moreover, off-gas analysis provided real-time information on biomass concentration as well as on cellular activity. Thus, the ideal time to start the production phase can be determined without the usual offset between sampling and analysis. The recording of pH and consumption of acid and base indicated the start of oil hydrolysis and the depletion of fatty acids towards the end of the process. Overall, these conclusions can be used to develop automated process control strategies based on real-time online values and to develop an even more robust process with hindsight for scale-up.

Another common observation was the formation of so-called MEL beads in the culture broth during production phase. These are globular aggregates of MEL, fatty acids and water that have been described before when *M. aphidis* was used as the production organism (Rau et al., 2005a; Goossens et al., 2016). Their formation was observed when a certain MEL concentration and ratio of MEL to substrate lipids (oil and fatty acids) was reached. Oil addition could dissolve these globules, but they were re-appearing when oil was hydrolyzed again. Formation of MEL beads interfered with sampling during production phase, as they were sticking to probes, baffles and the reactor wall. This could be the reason for variation in offline-measured data during production phase, for example, when a slight decrease in MEL concentration was noted towards the end of the process.

The ratio of the four MEL variants MEL-A,-B,-C and–D, which is characteristic for each producer species, was relatively constant over the time course of the fermentations and also between runs. An average MEL composition of 45 ± 2% MEL-A, 21 ± 1% MEL-B, 23 ± 3% MEL-C and 11 ± 3% MEL-D was calculated over all fermentation processes. Analysis of the acyl side-chains in the MEL yielded predominantly C_8_ and C_10_ acids. Overall, this is the characteristic composition of *M. aphidis* MEL, which has also been described in prior publications with this strain (Rau et al., 2005b; Onghena et al., 2011; Beck et al., 2019a)

HPLC analysis of the culture supernatants showed an increase in mannitol concentration during production phase, which has so far never been described for a MEL production process. In the fed-batch fermentations, an increase up to 35 g/L mannitol was detected. Mannitol accumulation in the broth was almost linear and correlated with oil hydrolysis. As long as hydrolysis was active and oil and fatty acids were remaining in the broth, like in FB1 and FB2, mannitol accumulated constantly. Only towards the end of the process and when all remaining fatty acids were consumed, like in FB3, mannitol concentration decreased again. We assume that mannitol is a side-product of the lipid metabolism in *M. aphidis*, as several studies have already shown that the production of polyols, like erythritol and mannitol, is a common feature in yeast (Carly and Fickers, 2018; Goncalves et al., 2019) and even in the related fungus *U. maydis* (Guevarra and Tabuchi, 1990). Ultimately, the production and accumulation of mannitol as a metabolic side-product is most likely also the reason for the occurrence of mannosylmannitol lipids (MML) along with MELs in certain Ustilaginaceae species, which has been reported by our group in a previous publication (Beck et al., 2019a).

### Kinetic Modeling of the MEL Fermentation Process

Ultimately, a knowledge-based model was developed and adjusted to our experimental data to gain a better understanding of the process kinetics. The partial models for cell growth and MEL production phase are based on a system of coupled ordinary differential equations (ODEs) that were solved numerically.

For the biomass growth phase, the concentration changes of glucose, sodium nitrate and biomass are described by a feeding term and Monod kinetics with glucose and nitrate as two non-complementary limiting substrates according to Roels (1983). The specific growth rate is dominated by the most limiting compound using a minimum operator (Eq 12).

$$µ={µ}_{max}\;min\left\{\frac{\;c_{Gluc}\;}{\left(K_{Gluc}+c_{Gluc}\right)\;};\frac{\;c_{NaNO3}}{\left(K_{NaNO3}+c_{NaNO3}\right)}\right\}$$ (12)

A possible growth inhibition at high substrate concentrations was not included in the model, as the currently used concentrations are assumed to be below this level. The changes in biomass (c_x_) and substrate (c_gluc_ and c_NaNO3_) concentrations are described by Eqs. 13–15:

$$\frac{dc_{X}}{dt}=-\frac{F_{in}}{V_{L}}c_{x}+µc_{X}$$ (13)

$$\frac{dc_{gluc}}{dt}=\frac{F_{in}}{V_{L}}\;\left(c_{gluc,Feed}-c_{gluc}\right)-q_{gluc}c_{x};with\;q_{gluc}=\frac{µ}{Y_{X/gluc}}$$ (14)

$$\frac{dc_{NaNO3}}{dt}=\frac{F_{in}}{V_{L}}\;\left(c_{NaNO3,Feed}-c_{NaNO3}\right)-q_{NaNO3}c_{x};with\;q_{NaNO3}=\frac{µ}{Y_{X/NaNO3}}$$ (15)

For the production phase, the concentration changes of biomass, oil, fatty acids and MEL are described by a feeding and three reaction terms in the respective model equations. All reaction terms depend on specific reaction rates q_n_ and the biomass concentration at the end of the growth phase (c_x,growth_), thus enabling to simulate the effect of an increased biomass concentration during growth phase on the consecutive production phase. The specific reaction rates q_n_ are based on Michaelis-Menten kinetics with maximum reaction rates q_max,n_ and substrate affinity constants K_m,n_ to represent the influence of decreasing substrate concentrations on the actual reaction rates. The first reaction term (q_hydrolysis_) represents the hydrolysis of oil/triglycerides into fatty acids and glycerol (Eq 16). A product inhibition constant K_i,hydrolysis_ was included to consider the lipase inhibition by high fatty acid concentrations (compare Henkel et al. (2014)). The second reaction term (q_MEL_) describes the conversion of fatty acids into MEL (Eq 17). Hence, the MEL production was simplified into a single “black-box” reaction term in a first approximation, although the MEL pathway in *M. aphidis* consists of many sequential steps in reality (Hewald et al., 2006; Lorenz et al., 2014; Günther et al., 2015). The third reaction term represents the accumulation of lipids within the cells, which removes fatty acids from the broth and converts them into intracellular lipid vesicles, thus increasing the cell biomass concentration (Eq 18). This was realized by simulating two types of “cell biomasses”, one that was lipid-free and generated during growth phase without nitrogen limitation (c_x,lipid-free_), and one that contains lipid vesicles (c_x,incl_) generated during production phase (see Eqs. 22, 23).

$$q_{hydrolysis}=q_{\;max,hydrolysis}\frac{\;c_{oil}}{c_{oil}+K_{m,hydrolysis}\left(1+\frac{c_{FA}}{K_{i,hydrolysis}}\right)}$$ (16)

$$q_{MEL}=q_{\;max,MEL}\frac{\;c_{FA}}{c_{FA}+K_{m,MEL}}$$ (17)

$$q_{incl}=q_{\;max,incl}\frac{\;c_{FA}}{c_{FA}+K_{m,incl}}$$ (18)

The changes in oil, fatty acid and MEL concentrations during the production phase are thus described by Eqs. 19–21:

$$\frac{dc_{Oil}}{dt}=\frac{F_{in}}{V_{L}}\left(c_{Oil,Feed}\;-c_{Oil}\right)-q_{hydrolysis}\;c_{x,growth}$$ (19)

$$\frac{dc_{FA}}{dt}=\;\frac{F_{in}}{V_{L}}\left(c_{FA,Feed}\;-c_{FA}\right)+\;Y_{FA/oil}\;q_{hydrolysis}\;c_{x,growth}-Y_{FA/MEL}\;\;q_{MEL}\;c_{x,growth}\;-Y_{FA/X,prod\;}\;q_{incl}\;c_{x,growth}$$ (20)

$$\frac{dc_{MEL}}{dt}=\;\frac{F_{in}}{V_{L}}\left(0\;-c_{MEL}\right)+\;q_{MEL}\;c_{X}$$ (21)

The changes in biomass concentrations, modeled as a decrease in c_x,lipid-free_ and an increase in c_x,incl_ by inclusion of fatty acids, is described by the following equations:

$$\frac{dc_{X,incl}}{dt}=\;\frac{F_{in}}{V_{L}}\left(0\;-c_{X,incl}\right)+\;q_{incl}c_{X,lipid-free}$$ (22)

$$\frac{dc_{X,lipid-free}}{dt}=\frac{F_{in}}{V_{L}}\left(0-c_{X,lipid-free}\right)+Y_{X,lipid-free/X,incl}q_{incl}\;c_{X,lipid-free}$$ (23)

The necessary input parameters for the different model equations were yield coefficients, biomass-specific rate constants and substrate affinity constants. A summary of all parameters used for kinetic modeling are presented in Table 4. The yield coefficients and biomass-specific rates were calculated from our experimental data. For example, biomass yield coefficients from glucose Y_X/Gluc_ and from sodium nitrate Y_X/NaNO3_ were derived from the amount of consumed substrate versus produced biomass using linear regression of process data. Biomass-specific consumption rates q_n_ were determined from a regression of substrate consumption rates r_n_ versus respective biomass concentration c_x_. The underlying assumption was that the biomass-specific rates and yield coefficients were constant during the process, which was in agreement with the experimental results. The yield coefficient Y_FA/oil_ was based on the reaction stoichiometry of oil hydrolysis. For simplification, “oil” was herein assumed as triolein, yielding 3 mol of oleic acid and 1 mol of glycerol during hydrolysis. The yield coefficient of biomass with lipid inclusions from fatty acids (Y_FA/X,incl_), as well as the ratio of lipid-free biomass during growth to biomass with lipid inclusions during production phase (Y_X,lipid-free/X,incl_) were based on a reaction stoichiometry that included the elementary compositions of the biomass during the different process stages shown in Table 1. The yield coefficient Y_FA/MEL_ for the formation of MEL from fatty acids could not be obtained directly from experimental data, since fatty acids are an intermediate product and their concentration depends on hydrolysis, lipid inclusion and MEL production simultaneously. Consequently, Y_FA/MEL_ was approximated to fit the model curves to our experimental data. The substrate affinity constants were also approximated to match the kinetic model curves to experimental data. The feeding terms in the simulations were set according to the experimental feeding rates in the respective processes.

**TABLE 4:** Overview of parameters used for kinetic modeling. Parameters were either calculated from experimental data, derived from reaction stoichiometry or approximated to fit the model to experimental data.

Partial Model	Parameter	Value	Based on
**Growth**	**µ**_**max**_ **(h** ^**−1**^ **)**	0.11	experimental data
	**q**_**max, gluc**_ **(g/gh)**	0.647	experimental data
	**K**_**m,Gluc**_ **(g/L)**	1	approximated
	**Y**_**X/Gluc**_ **(g/g)**	0.17	experimental data
	**q**_**max, NaNO3**_ **(g/gh)**	0.065	experimental data
	**K**_**m,NaNO3**_ **(g/L)**	0.01	approximated
	**Y**_**X/NaNO3**_ **(g/g)**	1.7	experimental data
**Production**	**q**_**max, hydrolysis**_ **(g/gh)**	0.1	experimental data
	**K**_**m,hydrolysis**_ **(g/L)**	5	approximated
	**K**_**i,hydrolysis**_ **(g/L)**	20	approximated
	**Y**_**FA/oil**_ **(g/g)**	0.957	stoichiometry
	**q**_**max,MEL**_ **(g/gh)**	0.02	experimental data
	**K**_**m,MEL**_ **(g/L)**	5	approximated
	**Y**_**FA/MEL**_ **(g/g)**	1.667	experimental data
**Lipid accumulation**	**q**_**incl,max**_ **(g/gh)**	0.07	approximated
	**K**_**m, incl**_ **(g/L)**	1	approximated
	**Y**_**X,unlim/X,incl**_ **(g/g)**	0.203	stoichiometry
	**Y**_**FA/X,incl**_ **(g/g)**	1.128	stoichiometry

**FIGURE 7:**
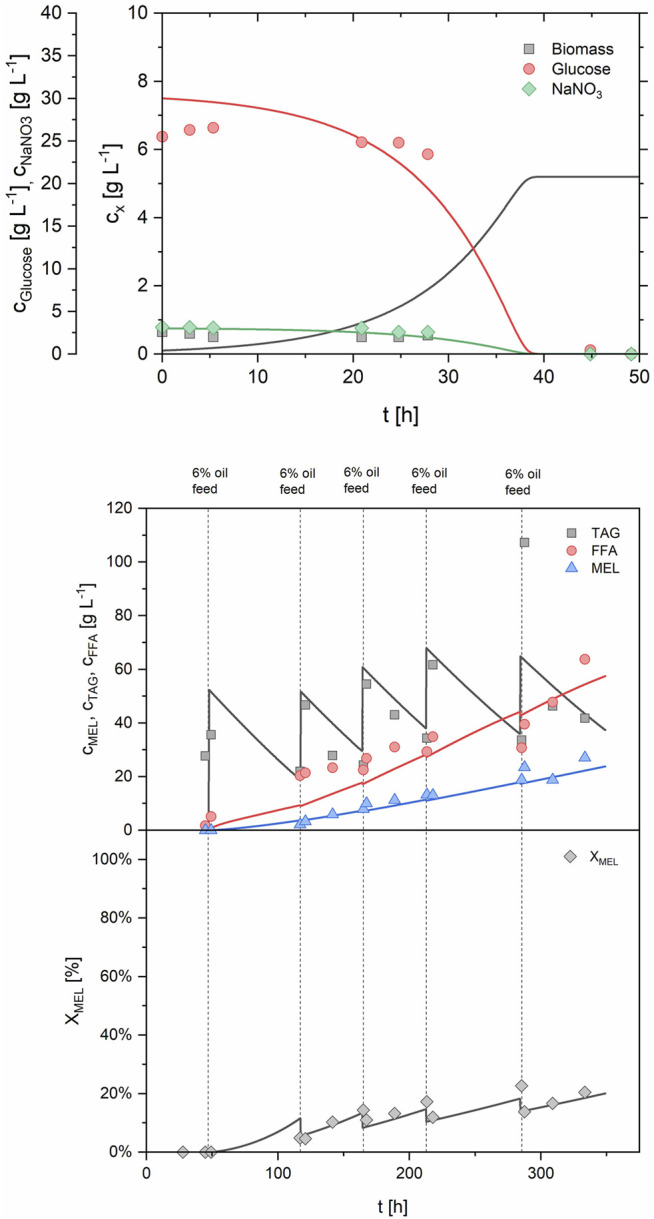
Comparison of simulated and experimental data for process B2 using the parameters from Table 4. Batch growth phase (upper panel) and production phase with repeated oil feeding (lower panel) are shown.

The developed model was able to simulate the time-course of biomass and substrate concentrations during batch and fed-batch growth, as well as the substrate and product concentrations during MEL production phase. The process B2 with batch growth phase and multiple oil feeds was used to approximate the missing model parameters as described before and to demonstrate the general correlation between the model and experimental data (Figure 7). A MEL concentration of 22.2 g/L and a X_MEL_ of 18.5% was predicted after 333 h, which was in reasonable agreement with the experimental values of 27.1 g/L and 20.4%.

**FIGURE 8:**
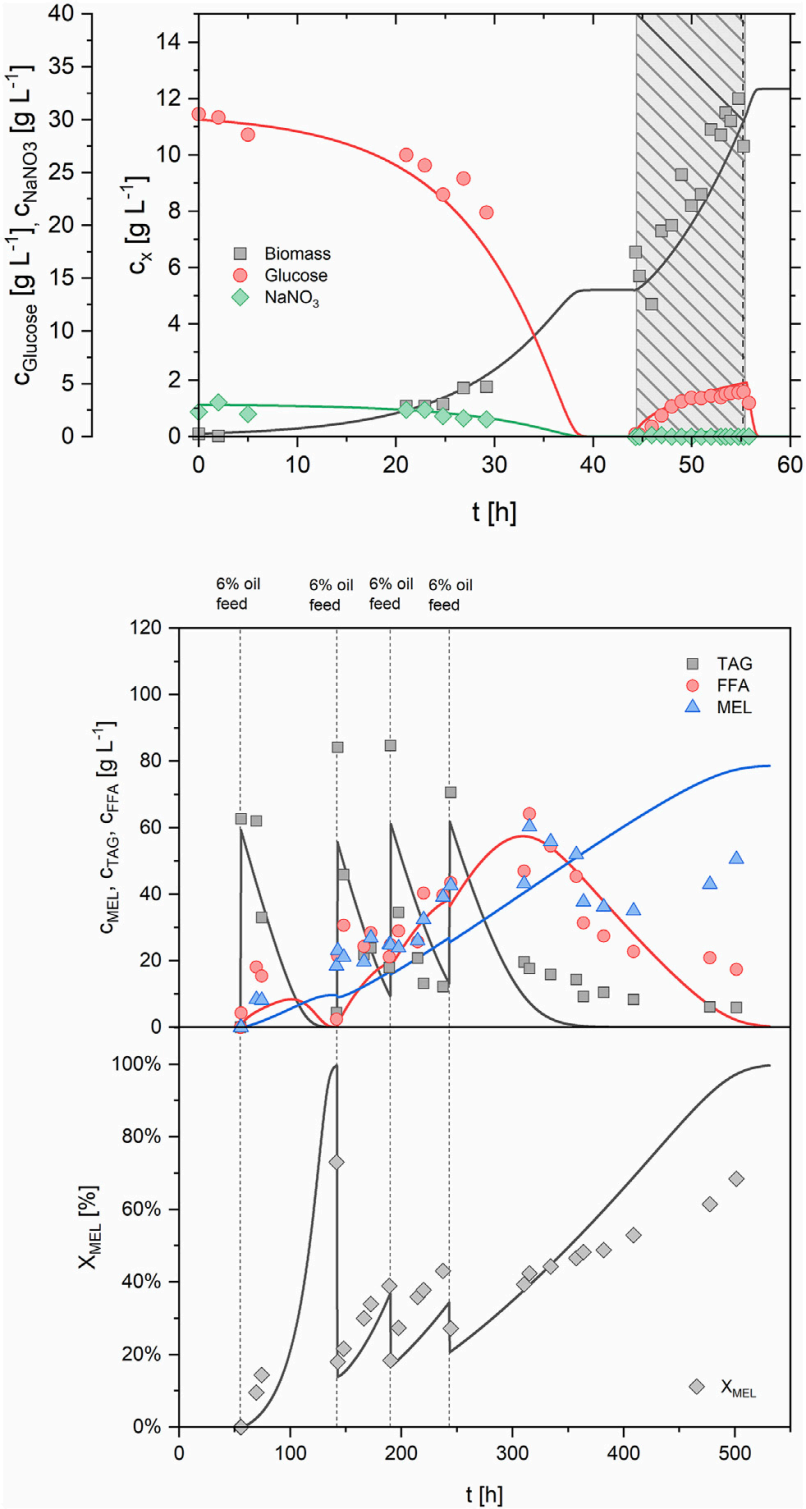
Comparison of simulated and experimental data for process FB1 using the parameters from Table 4. Batch and fed-batch growth phase (upper, feeding phase is highlighted) and production phase with repeated oil feeding (lower) are shown.

After all parameters were adjusted to the data of B2, the model was used to simulate the process FB1 with batch and fed-batch growth phase, employing the same feeding strategy as in the experiment. The growth model predicted a biomass concentration of 12.3 g/L at the end of the fed-batch phase, compared to 12.0 g/L from the experiment (Figure 8). As expected, the reaction rates during production phase were automatically increased according to the increased c_x,growth_ in the model, and correlated well with experimental data. A MEL concentration of 40.4 g/L and a X_MEL_ of 37.6% were predicted after 310 h, compared to 43.2 g/L and 39.3% in the experiment. After 350 h, however, larger deviations between model and experimental data occurred, which could be explained by a decreased activity of lipases and cellular metabolism, which is however not included in the model. Another explanation might be the formation of MEL beads or the deposition of MEL and other hydrophobic substances at the reactor vessel and baffles, as described before. In general, it should be noted that the developed model is only valid within certain limits that are determined by the model restraints.

Overall, this is the first time that a kinetic model based on a system of ordinary differential equations has been presented for MEL production. Previously, there were only kinetic models for other biosurfactants like sophorolipids (García-Ochoa and Casas, 1999) and rhamnolipids (Henkel et al., 2014). The rhamnolipids production model by Henkel et al. (2014) also employs a complex system of differential equations describing the cellular growth on different substrates, oil hydrolysis and rhamnolipids production. Since this is relatively similar to the MEL production process, it served as a reference for developing our model. The advantage of the here presented model is the possibility to evaluate different feeding strategies for medium and oil during growth and MEL production phase, respectively. A possible limitation of the model is the strict separation between growth and production phase models, assuming that the cells have entered stationary phase when production is started. Nevertheless, the current model is able to describe the MEL production process in detail and will be used to further optimize and evaluate MEL production.

## Conclusion

The process for MEL production in an aerated stirred-tank bioreactor with *M. aphidis*, using a previously developed mineral salt medium, has been evaluated and enhanced by establishing an exponential fed-batch growth phase. Several experiments in the aerated STBR have shown that exponential substrate feeding of a concentrated solution containing glucose and nitrate led to a 2-3-fold increase of biomass concentration compared with a batch process. The substrate feeding, which was started after an initial batch growth phase, was conducted with defined growth rates of 0.08 or 0.09 h^−1^ and led to increased biomass concentrations of 10.9–15.5 g/L, compared with 4.2 g/L in the batch process. In the first two of the presented fed-batch processes, MEL production phase was conducted using 16–20 g_oil_/g_biomass_. This led to high MEL concentrations of 50.5 or 43.9 g/L, but at the same time to high concentrations of residual oil and fatty acids. Thus, a low MEL percentage in the crude lipid extract of 68 and 49% was obtained, respectively, even after long process times of up to 501 h. Oil feeding was therefore reduced to around 9–11 g_oil_/g_biomass_ in the last two fed-batch processes, which in turn led to slightly lower concentrations of 35.7 and 34.3 g/L MEL. At the same time however, the MEL yields from oil were increased to 0.294 and 0.275 g/g in the optimized processes and the substrate lipid was almost fully converted into product, leaving only traces of fatty acids in the broth at the end of the process. The corresponding share of MEL in the crude extract was increased to 88 and 95% respectively, and process time could be reduced to around 170 h. The increased purity of the crude lipid extract has also positive implications for the subsequent downstream processing of the broth, since, for example, a simple solvent extraction might be sufficient to obtain a product with already satisfactory purity. Flash chromatography, which is commonly employed to remove remaining oil and fatty acids from the crude extract, can be omitted if the required purity is already obtained by biological means at the end of the fermentation process.

Foaming, which has always been a common and major issue in biosurfactant production processes, was reduced by using the mineral salt medium and mechanical foam destruction in the headspace of the bioreactor, although it was still present at high biomass concentrations towards the end of the growth phase. Several online parameters like dissolved oxygen, pH trends and off-gas analysis correlated well with biomass growth, substrate consumption and product formation, and could be used for process monitoring and control. The MEL composition was shown to be typical for *M. aphidis* and the reproducibility between the different runs was very high.

Ultimately, kinetic model equations were established for simulation and prediction of process behavior during cell growth and MEL production phase. The model was able to represent the experimental data accurately. To the best of our knowledge, this is the first time that a kinetic model based on a system of ordinary differential equations has been developed and employed for the prediction of the MEL production process. This model will be used in the future to scale the production process and perform a life-cycle assessment and techno-economic analysis that will provide a better understanding of key economic and ecological parameters for MEL production.

## Data Availability

The original contributions presented in the study are included in the article/Supplementary Material, further inquiries can be directed to the corresponding author.
