# Supplementary material for: Optimization and Kinetic Modeling of a Fed-Batch Fermentation for Mannosylerythritol Lipids (MEL) Production With Moesziomyces aphidis
Source: Front Bioeng Biotechnol. 2022 May 17;10:913362. doi: 10.3389/fbioe.2022.913362 (PMC9152284; doi:10.3389/fbioe.2022.913362)
Supplement: Supplementary file 1 [file DataSheet1.DOCX]

Supplementary Material

# Supplementary Tables

Supplementary Table S 1: Experimental matrix for the Box-Behnken design to evaluate different medium compositions. Input variables (concentration of N, P and S) and output variables (biomass concentration, yield, MEL concentration, crude extract concentration and X_MEL_) are shown.

|  |  | **Input variables** | | |  | **Output variables** | | | | | | |
| --- | --- | --- | --- | --- | --- | --- | --- | --- | --- | --- | --- | --- |
| **Run** | **Std** | **c_NaNO3_  (g/L)** | **c_KH2PO4_  (g/L)** | **c_MgSO4*7H2O_  (g/L)** |  | **c_x,growth_ ^*^ (g/L)** | **Y_X/Gluc_  (g/g)** | **c_MEL_  (g/L)** | **c_crude extract_  (g/L)** | **X_MEL_  (%)** | |  |
| 1 | 2 | 6 | 1 | 1.5 |  | 4.4 | 0.15 | 5.7 | 7.7 | 74% | |  |
| 2 | 14 | 4.5 | 1.5 | 1.5 |  | 4.5 | 0.15 | 11.0 | 14.0 | 79% | |  |
| 3 | 4 | 6 | 2 | 1.5 |  | 4.9 | 0.16 | 7.4 | 9.2 | 80% | |  |
| 4 | 10 | 4.5 | 2 | 1 |  | 4.6 | 0.15 | 9.3 | 11.0 | 84% | |  |
| 5 | 13 | 4.5 | 1.5 | 1.5 |  | 4.8 | 0.16 | 12.2 | 16.4 | 74% | |  |
| 6 | 3 | 3 | 2 | 1.5 |  | 5.4 | 0.18 | 15.8 | 18.8 | 84% | |  |
| 7 | 6 | 6 | 1.5 | 1 |  | 4.6 | 0.15 | 8.7 | 11.1 | 79% | |  |
| 8 | 9 | 4.5 | 1 | 1 |  | 4.7 | 0.16 | 13.4 | 17.3 | 78% | |  |
| 9 | 5 | 3 | 1.5 | 1 |  | 5.7 | 0.19 | 15.6 | 18.2 | 86% | |  |
| 10 | 15 | 4.5 | 1.5 | 1.5 |  | 4.6 | 0.15 | 9.8 | 12.2 | 81% | |  |
| 11 | 11 | 4.5 | 1 | 2 |  | 4.3 | 0.14 | 9.2 | 13.6 | 68% | |  |
| 12 | 12 | 4.5 | 2 | 2 |  | 4.8 | 0.16 | 9.0 | 13.0 | 69% | |  |
| 13 | 7 | 3 | 1.5 | 2 |  | 5.4 | 0.18 | 16.4 | 20.5 | 80% | |  |
| 14 | 1 | 3 | 1 | 1.5 |  | 5.2 | 0.17 | 26.1 | 31.6 | 83% | |  |
| 15 | 8 | 6 | 1.5 | 2 |  | 4.7 | 0.16 | 7.3 | 9.3 | 79% | |  |
| 16 | Verification low | 3 | 1 | 1 |  | 5.5 | 0.18 | 21.7 | 28.4 | 77% | |  |
| 17 | Verification high | 6 | 2 | 2 |  | 5.0 | 0.17 | 4.8 | 6.2 | 77% | |  |
|  |  |  |  |  |  |  |  |  |  |  | |  |
| * from correlation of backscatter values with dry biomass concentration | | | | | | | | | | |  |  |

Supplementary Table S 2: Results of the sugar substrate screening. All sugars were applied at a total sugar concentration of 30 g/L to ensure comparability.

| **substrate** | **c_x,growth_ ^*^ (g/L)** | **Y_X/Gluc_  (g/g)** | **c_MEL_  (g/L)** | **c_crude extract_  (g/L)** | **X_MEL_  (%)** |
| --- | --- | --- | --- | --- | --- |
| glucose | 4.6 | 0.15 | 15.7 | 21.6 | 73% |
| arabinose | 3.4 | 0.11 | 10.1 | 49.1 | 28% |
| cellobiose | 0.6 | 0.02 | 12.0 | 26.2 | 38% |
| sugar beet molasses | 4.8 | 0.16 | 9.1 | 24.7 | 49% |
| syrup | 4.0 | 0.13 | 8.7 | 27.5 | 38% |
| sugar cane molasses | 2.4 | 0.08 | 13.9 | 44.9 | 31% |
| sucrose | 4.6 | 0.15 | 19.5 | 31.7 | 61% |
| xylose | 3.0 | 0.10 | 19.9 | 24.7 | 81% |
| fructose | 3.8 | 0.13 | 21.1 | 25.6 | 82% |
| process water B | 4.4 | 0.15 | 23.4 | 28.9 | 81% |
| process water A | 4.4 | 0.15 | 16.3 | 20.9 | 78% |

Supplementary Table S 3: Average MEL composition for the different process runs and overall mean values.

|  |  | **MEL-A (%)** | **MEL-B (%)** | **MEL-C /  MML-A (%)** | **MEL-D /  MML-B/C (%)** |
| --- | --- | --- | --- | --- | --- |
| B1 | mean | 47.2 | 19.5 | 26.3 | 7.1 |
|  | SD | 2.8 | 0.5 | 2.5 | 0.4 |
| B2 | mean | 38.9 | 12.4 | 40.4 | 8.3 |
|  | SD | 7.0 | 4.1 | 2.2 | 3.1 |
| FB1 | mean | 47.1 | 21.7 | 20.6 | 11.6 |
|  | SD | 7.9 | 2.8 | 2.3 | 2.1 |
| FB2 | mean | 47.8 | 19.0 | 23.3 | 9.9 |
|  | SD | 9.2 | 3.2 | 4.2 | 2.3 |
| FB3 | mean | 43.4 | 20.6 | 25.3 | 14.0 |
|  | SD | 7.9 | 5.7 | 6.1 | 2.7 |
| FB4 | mean | 43.5 | 22.5 | 18.4 | 15.5 |
|  | SD | 5.8 | 3.3 | 3.2 | 2.0 |
| Overall average | mean | 45.8 | 20.7 | 22.8 | 11.6 |
|  | SD | 2.14 | 1.47 | 3.25 | 3.34 |

# Supplementary Figures

|  |  |
| --- | --- |

Supplementary Figure S 1: Process data for the two-staged batch with repeated oil feeding (B2). Growth was performed in batch mode, and production was initiated with 6 % rapeseed oil at 42 h and maintained using multiple oil feeds of 4 % at 118, 165, 214 and 286 h.

|  |  |
| --- | --- |

Supplementary Figure S 2: Process data for process FB1. Growth was performed in batch and additional fed-batch mode with an exponential feed rate at µ_set_ = 0.08 h^-1^, and production was initiated and maintained using multiple oil feeds of 6 % at 56, 142, 190 and 242 h.

|  |  |
| --- | --- |

Supplementary Figure S 3: Process data for process FB2. Growth was performed in batch and additional fed-batch mode with an exponential feed rate at µ_set_ = 0.09 h^-1^, and production was initiated with 6 % rapeseed oil at 55 h and a continuous oil feed between 71-141 h (6 % in total).

|  |  |
| --- | --- |

Supplementary Figure S 4: Process data for process FB3. Growth was performed in batch and additional fed-batch mode with an exponential feed rate at µ_set_ = 0.09 h^-1^, and production was initiated and maintained using two separate oil feeds of 6% at 51 and 72 h.

|  |  |
| --- | --- |

Supplementary Figure S 5: Process data for process FB4. Growth was performed in batch and additional fed-batch mode with an exponential feed rate at µ_set_ = 0.08 h^-1^, and production was initiated using 6 % rapeseed oil at 44 h and a continuous feeding between 52-82 h (6% in total).
